# Correlations of receptor desensitization of gain-of-function *GABRB3* variants with clinical severity

**DOI:** 10.1093/brain/awad285

**Published:** 2023-08-30

**Authors:** Susan X N Lin, Philip K Ahring, Angelo Keramidas, Vivian W Y Liao, Rikke S Møller, Mary Chebib, Nathan L Absalom

**Affiliations:** Brain and Mind Centre, School of Medical Sciences, Faculty of Medicine and Health, The University of Sydney, Sydney, New South Wales 2006, Australia; Brain and Mind Centre, School of Medical Sciences, Faculty of Medicine and Health, The University of Sydney, Sydney, New South Wales 2006, Australia; Institute for Molecular Bioscience, The University of Queensland, Saint Lucia, QLD 4072, Australia; Brain and Mind Centre, School of Medical Sciences, Faculty of Medicine and Health, The University of Sydney, Sydney, New South Wales 2006, Australia; Department of Epilepsy Genetics and Personalized Medicine, Member of ERN, EpiCare, Danish Epilepsy Centre, Dianalund DK-4293, Denmark; Department of Regional Health Research, Faculty of Health Sciences, University of Southern Denmark, Odense DK-5230, Denmark; Brain and Mind Centre, School of Medical Sciences, Faculty of Medicine and Health, The University of Sydney, Sydney, New South Wales 2006, Australia; Brain and Mind Centre, School of Medical Sciences, Faculty of Medicine and Health, The University of Sydney, Sydney, New South Wales 2006, Australia; School of Science, University of Western Sydney, Sydney, New South Wales, Australia

**Keywords:** GABA_A_ receptor, developmental and epileptic encephalopathy, electrophysiology, EIMFS, movement disorder, *SLC12A5*

## Abstract

Genetic variants associated with developmental and epileptic encephalopathies have been identified in the *GABRB3* gene that encodes the β3 subunit of GABA_A_ receptors. Typically, variants alter receptor sensitivity to GABA resulting in either gain- or loss-of-function, which correlates with patient phenotypes. However, it is unclear how another important receptor property, desensitization, contributes to the greater clinical severity of gain-of-function variants.

Desensitization properties of 20 gain-of-function *GABRB3* variant receptors were evaluated using two-electrode voltage-clamp electrophysiology. The parameters measured included current decay rates and steady-state currents. Selected variants with increased or reduced desensitization were also evaluated using whole-cell electrophysiology in transfected mammalian cell lines.

Of the 20 gain-of-function variants assessed, 13 were found to alter receptor desensitization properties. Seven variants reduced desensitization at equilibrium, which acts to worsen gain-of-function traits. Six variants accelerated current decay kinetics, which limits gain-of-function traits. All affected patients displayed severe clinical phenotypes with intellectual disability and difficult-to-treat epilepsy. Nevertheless, variants that reduced desensitization at equilibrium were associated with more severe clinical outcomes. This included younger age of first seizure onset (median 0.5 months), movement disorders (dystonia and dyskinesia), epilepsy of infancy with migrating focal seizures (EIMFS) and risk of early mortality. Variants that accelerated current decay kinetics were associated with slightly milder phenotypes with later seizure onset (median 4 months), unclassifiable developmental and epileptic encephalopathies or Lennox–Gastaut syndrome and no movement disorders.

Our study reveals that gain-of-function *GABRB3* variants can increase or decrease receptor desensitization properties and that there is a correlation with the degree of disease severity. Variants that reduced the desensitization at equilibrium were clustered in the transmembrane regions that constitute the channel pore and correlated with greater disease severity, while variants that accelerated current decay were clustered in the coupling loops responsible for receptor activation and correlated with lesser severity.

## Teaser

Lin *et al.* show that pathogenic *GABRB3* variants can either increase or decrease desensitization of GABA_A_ receptors. Reduced receptor desensitization at equilibrium is associated with more severe clinical outcomes, including early mortality, younger age of seizure onset, and epilepsy of infancy with migrating focal seizures.

## Introduction

Developmental and epileptic encephalopathy (DEE) consists of a heterogeneous group of genetic epileptic disorders that begin early in childhood and are associated with neurological impairment, with many patients refractive to treatment.^1^ Missense *de novo* variants in γ-aminobutyric acid type A (GABA_A_) receptor subunit-encoding genes have recently been associated with DEE.^2^

GABA_A_ receptors are key mediators of neuronal inhibition, opening an intrinsic chloride channel in response to GABA release at inhibitory synapses to hyperpolarize the cell. Consistent with this role in neuronal inhibition, DEE-associated variants were reported to reduce chloride channel activity, with greater losses in receptor function proposed to increase clinical phenotype severity.^3-8^ However, we and others, have recently shown that GABA_A_ receptor variants not only cause loss-of-function, but also gain-of-function receptors.^9-12^ Importantly, gain- and loss-of-function *GABRB3* variants are associated with markedly different clinical outcomes, whereby patients with gain-of-function variants typically have more severe phenotypes.

**Figure 1:**
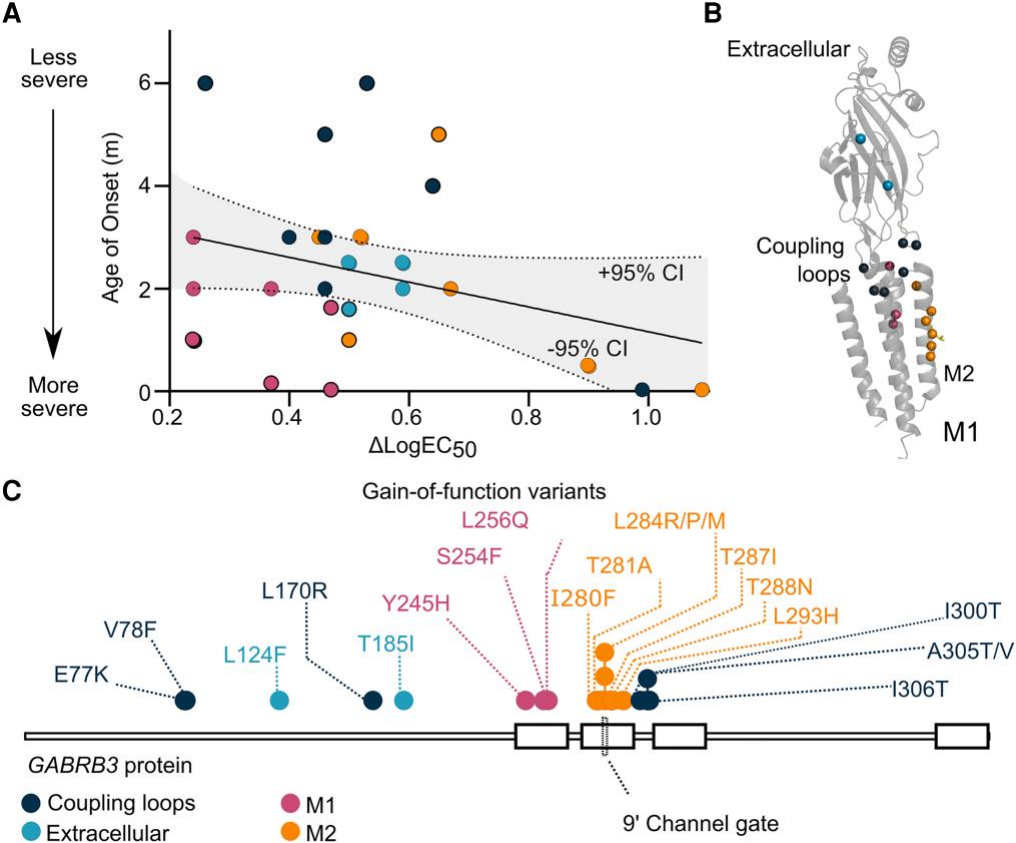
**Clinical and functional effects of gain-of-function variants located in distinct structural motifs**. (**A**) Linear regression of the age of seizure onset against the ΔlogEC_50_ at gain-of-function *GABRB3* variants. Values for 31 patients harbouring 20 variants were taken from Absalom *et al*.^9^ and Yang *et al*.^15^ The line of best fit and area within 95% confidence intervals are shown. Filled circles represent variants at residues in the coupling region (dark blue), extracellular region (light blue), M1 (magenta) and M2 (orange). Black outline denotes variants that are outliers in the linear regression of age of onset and ΔlogEC_50_. (**B**) Location of gain-of-function variants (spheres) within the cryogenic electron microscopy structure of β3 subunit of the GABA_A_ receptor (PDB:6HUP). (**C**) 2D representation of the protein sequence of the β3 subunit of the GABA_A_ receptor. Gain-of-function variants are represented as dots. M1 = M1 transmembrane helix; M2 = M2 transmembrane helix.

The *GABRB3* gene codes for the β3 subunit of GABA_A_ receptors, and a striking feature of variants in this and other GABA_A_ genes is the association between variant structural location and severity of the clinical phenotype, with transmembrane M1 and M2 helix variants most severe.^3,4,8,13,14^ Intuitively, clinical severity would be expected to be linked to the magnitude of observed functional change. However, a correlation plot of age of onset for first seizure event and receptor functional change for gain-of-function *GABRB3* variants does not reveal a good correlation (Fig. 1A). Seemingly irrespective of functional consequence, patients harbouring variants in the transmembrane M1 and M2 helices typically present with a very young age of onset (<3 months), and conversely those harbouring variants in the coupling regions and extracellular domain present with a later age of onset (>3 months) (Fig. 1A and B). It is unclear why the regional location of the variant holds such importance for clinical severity.

Structural motifs that harbour variants also influence receptor desensitization, whereby receptors enter a long-lived agonist bound closed state resistant to GABA activation. To date, only variants that increase desensitization by accelerating current decay have been reported.^16-19^ In some cases (e.g. γ2^R323Q^), desensitization exacerbated loss-of-function of receptors with impaired GABA sensitivity.^16^ In others (e.g. α5^V294L^ and α1^A332V^), accompanying increased GABA sensitivity resulted in mixed loss- and gain-of-function characteristics.^17,19^ Increased desensitization was proposed to remove activatable receptors from the available pool and effectively lower inhibitory tone, leading to de facto loss-of-function, but no empirical data measuring neuronal activity has shown that variants with increased desensitization lower the inhibitory tone.^17^ However, mutational studies have shown that single amino acid transmembrane domain mutations can either increase or decrease receptor desensitization.^20-25^ Hence, there is no *a priori* reason that DEE-associated variants should only affect desensitization in one way.

In this study, we hypothesized that changes in desensitization characteristics are one component that is associated with more severe clinical outcomes at transmembrane domain variants. To investigate this, we analysed 20 gain- and 4 loss-of-function *GABRB3* variants encompassing key structural motifs. We show that gain-of-function variants can have either increased or decreased receptor desensitization properties which correlates with the variant location and provide evidence suggesting that this property modulates clinical outcomes.

## Materials and methods

### Molecular biology

Concatenated receptors were created as previously described.^26,27^ Briefly, the γ2-β3-α1-β3-α1 concatenated construct was created with six unique restriction enzyme sites to enable efficient removal of wild-type (WT) subunits and insertion of variants. Variant subunits were purchased from Genscript and subcloned into the concatenated receptor. The resulting construct contained the subunits with linker sequences in the order of γ2-(AGS)_5_-β3-(AGS)_5_LGS(AGS)_3_-α1-AGT(AGS)_5_-β3-(AGS)_4_ATG(AGS)_4_-α1. The γ2 variant was subcloned into the first subunit, and β3 variants into the second. DNA gel electrophoresis was performed to ensure the incorporation of the five subunits. cRNA was produced from linearized cDNA using the mMessage mMachine™ T7 Transcription kit (Thermo Fisher) according to the manufacturer's description and stored at −20°C until use. WT and variant constructs containing the α1, β3 and γ2 subunits as previously described^27^ were purchased from Genscript.

### Oocyte expression and electrophysiology

Oocytes were obtained from an ovary segment of *Xenopus laevis* from the University of Wollongong under animal ethics protocol AE2003. Single oocytes were microinjected with ∼25 ng of either concatemeric GABA_A_ receptor cRNA, α1 and β3 cRNA in a 1:5 ratio or α1, β3 and γ2 cRNA in a 5:1:5 or 10:1:10 ratio and were then incubated for 1–3 days as previously described.^26,27^ Oocytes were impaled with 3 M KCl filled borosilicate glass microelectrodes, with a resistance of 0.2–1.6 MΩ, then voltage clamped at −60 mV. Oocytes were continuously perfused with ND96 buffer (in mM, 96 NaCl, 2 KCl, 1 MgCl_2_.6H_2_O, 5 HEPES hemisodium, 1.8 CaCl_2_) through a semi-automatic gravity-driven perfusion system at 1 ml/min. Solutions were applied via two separate tubes to remove any dead volume in the application of the test tube. Solutions were switched between bath and test solutions with pre-programmed electronic valves and the time between opening the valve and the peak current was in the range of 1–2 s. Currents were recorded using a Warner amplifier OC-725C and sampled at a frequency of 1 kHz and filtered at 10 Hz, then digitalized with LabChart reader version 8.1 (AD Instruments). All experiments were conducted at room temperature.

The recording protocol began with a 2-min wash with ND96 buffer, followed by an application of 3 mM GABA for 150 s, then a 5-min wash, 150 s 3 mM GABA application, 1-min wash, 20 s application of 10 mM GABA and 10 μM etomidate to prime the tubing. An 8-min wash with ND96 buffer ensued for receptors to return from the desensitized state, followed by a 120 s co-application of 10 mM GABA and 10 μM etomidate. To determine the maximum receptor open probability [Est P_O(max)_], the peak current elicited by the second 3 mM GABA application was normalized to the peak current elicited by 10 mM GABA and 10 μM etomidate. Etomidate was used to estimate the maximum open probability as receptors contain two etomidate binding sites that contribute equally and non-cooperatively to modulation.^28^ In the rare event that a variant affects the binding site, one etomidate site will be unchanged and available for modulation and estimation of maximum open probability. Current decay rate was calculated by analysis of the second 3 mM GABA application. The first 3 mM GABA application was used to compute the maximum open probability and current decay rate of β3^I300T^ variant, where excessive desensitization substantially reduced the peak current at the second GABA application. To ensure a saturating concentration of GABA was applied to loss-of-function variants, an additional experiment was performed where 30 mM instead of 3 mM GABA was applied, and 30 mM GABA and 10 μM etomidate was co-applied.

### HEK293 cell electrophysiology

HEK293AD cells were grown in Dulbecco’s modified Eagle medium with 10% fetal bovine serum until they reached about 80–90% confluency. The cells were then dissociated and plated onto 12 mm glass coverslips at a confluency of 50% 1 day prior to transfection. HEK293AD cells were transfected using the calcium phosphate co-precipitation method with separate cDNAs encoding the WT α1, β3 and γ2s subunits or the α1, β3^T287I^, γ2s or α1, β3 and γ2s^R323Q^ variant containing subunit combinations at a α:β:γ ratio of 1:1:3. The CD4 surface antigen was also included in the transfection mix and served as a marker for transfected cells. Standard whole-cell or outside-out patch clamp recordings were carried out 36–48 h post-transfection in standard extracellular solution containing (in mM). Unless otherwise indicated the experiments were carried out at a clamped potential of −70 mV. An EPC 10 USB Heka Patch Clamp Amplifier (HEKA, Elekronik). Currents were filtered (−3 dB, 4-pole Bessel) at 5 kHz and sampled at 50 kHz and PatchMaster software were used to record whole-cell and macropatch currents. Patch electrodes were fabricated from borosilicate glass capillaries (G150F-3; Warner Instruments) and heat-polished to a final resistance of 3–6 MΩ when filled with intracellular solution. The intracellular solution contained (in mM) 145 CsCl, 2 MgCl_2_, 2 CaCl_2_, 10 HEPES and 5 EGTA, and adjusted to pH 7.4 with CsOH. The extracellular solution was composed of (in mM) 140 NaCl, 5 KCl, 2 CaCl_2_, 1 MgCl_2_, 10 HEPES and 10 D-glucose, and adjusted to pH 7.4 with NaOH. Currents were elicited by rapid application of 3 mM GABA via a double-barrelled glass tube that was mounted onto a piezo-electric translator (Siskiyou). This method achieved a solution exchange time of ∼1 ms over small cells (15–20 pF) or outside-out membrane patches by lateral movement of the glass tube.^29,30^ Data from small cells and patches were pooled. To determine receptor desensitization and steady-state currents, GABA was applied for several seconds, whereas to determine current activation and deactivation times, GABA was applied for ∼1–2 ms.

### Data analysis

Linear and non-linear regression was performed with GraphPad Prism version 8.0.1. Current decay rates were digitized using LabChart and analysed with Microsoft Excel and GraphPad Prism. Traces were fitted to a one-phase exponential decay equation:


(1) $$Y=(Y0-Plateau)\times e^{-kX}\;+Plateau$$

Where *Y* and *X* are the amplitude and time, respectively. The plateau, or the asymptote of the each fitted trace, is the current amplitude at infinite time or steady-state (*I*_ss_) and k (s^−1^) is the rate constant of current decay. To define the maximum steady-state open probability [Est P_O(ss,max)_], the *I*_ss_ was normalized to the 3 mM GABA current and then transformed to the Est P_O(max)_ determined for each day of recording at a specific construct.

Subsequently, a two-phase exponential decay was also fitted to traces using the equation:


(2) $$\begin{array}{rl} Y= & \;(Y0-Plateau)\times Fraction_{Fast}\times e^{-X/\tau_{Fast}\;}\\ & +(Y0-Plateau)\times Fraction_{Slow}\times e^{-X/\tau_{Slow}}+Plateau \end{array}$$

The weighted time constant (τ_weighted_) was determined by the following equation


(3) $$\tau_{\mathrm{weighted}}=Fraction_{Fast}\times \tau_{\mathrm{fast}}+\;Fraction_{Slow}\times \tau_{\mathrm{Slow}}\;$$

### Statistical analysis

All analysis was performed using GraphPad Prism version 8. One-way ANOVA (Kruskal–Wallis rank sum test) was performed to make comparisons between groups followed by a Dunn’s *post hoc* test. Variants were split into four groups of equal size and comparisons were made between variants and WT receptors that were recorded on the same day. Where the Kruskal–Wallis test failed to show a significant difference between groups (*P* > 0.05), Dunn’s *post hoc* test was not applied. Values for the Kruskal–Wallis statistic, degrees of freedom and *P*-value can be found in the Supplementary material. A Mann–Whitney U-test was performed to compare parameters where only one variant was compared to the WT and is indicated in the tables. A one-way ANOVA with Dunnett’s *post hoc* test was performed to compare the logEC_50_ of concatenated and untethered receptors. Oocytes with a holding potential less than −200 nA were excluded from the study, with the exception of variants known to induce constitutive activity (e.g. β3^L284R^). For each experiment at least two batches of oocytes were used. All data are presented as mean ± standard deviation (SD).

## Results

We previously described clinical outcomes for patients harbouring gain- and loss-of-function *GABRB3* variants based on changes in GABA sensitivity at GABA_A_ receptors.^9^ For this study, we omitted protein truncating variants and supplemented our previous cohort of 27 gain and 37 loss-of-function patients with a recent study that outlined details of six patients harbouring *GABRB3* variants (one loss- and five gain-of-function) with known changes in the GABA sensitivity.^9,15^ Data from additional patients and follow up of previously reported patients reinforced the previous findings of the overall clinical phenotypes. Patients with gain-of-function *GABRB3* variants presented with a median age of first seizure onset of 2 months [95% confidence interval (CI) 1.6–3], severe to profound intellectual disability (ID) (23/32 patients), epilepsy syndromes described predominantly as unclassified DEEs (16/32), or epilepsy of infancy with migrating focal seizures (EIMFS) (9/32), microcephaly (13/32) and movement disorders including dystonia and dyskinesia (10/32) (Table 1). In contrast, patients with loss-of-function variants presented with a median age of first seizure onset of 10 months (95% CI 8–15), fewer cases of severe ID (8/38 patients), a spectrum of different epilepsy syndromes, no movement disorders except for ataxia (5/38), no microcephaly (0/38) and little reported early mortality (1/38).

**Table 1:** Clinical parameters of patients with *GABRB3* variants

Functional consequence	All LOF	All GOF	GOF			
Desensitization property			No changes to desensitization	Accelerated current decay	Decreased desensitization at equilibrium	Accelerated current decay and decreased desensitization at equilibrium
*n*	38	32	10	8	10	4
Age at seizure onset, months (median ± CI)	10 (8–15)	2 (1.6–3)	2.5 (1.6–3)	4 (2–6)	0.5 (0.03–3)	1.5 (0.5–3)
Epilepsy syndromes	GEFS+ (4) Focal epilepsy (4) EMAS (1) DEE (7) West syndrome (3) Dravet (5) MAE (3) LGS (4) Unclassified (6)	DEE (16) EIMFS (9) West syndrome (2) LGS (2) Unclassified (3)	DEE (7) EIMFS (3)	DEE (5) LGS (2) Unclassified (1)	DEE (2) EIMFS (4) West syndrome (2) Unclassified (2)	DEE (2) EIMFS (2)
Level of cognition	Normal (1) GDD (6) Mild ID (11) Moderate ID (12) Severe ID (8)	GDD (8) Moderate ID (1) Severe/profound ID (23)	GDD (2) Severe/profound ID (8)	GDD (1) Severe/profound ID (7)	GDD (2) Moderate ID (1) Severe/profound ID (7)	GDD (3) Severe/profound ID (1)
Movement disorders and types	5/38 Ataxia (5)	10/32 Dystonia (5) Dyskinesia (3) Not specified (2)	4/10 Dystonia (2) Dyskinesia (1) Not specified (1)	0/8	5/10 Dystonia (3) Dyskinesia (1) Not specified (1)	1/4 Dyskinesia (1)
Microcephaly	0/38	13/32 (40,6%)	4/10 (40%)	3/8 (37,5%)	4/10 (40%)	2/4 (50%)
Mortality	1/38 (2,6%)	5/32 (15,6%)	2/10 (20%)	0/8	3/10 (30%)	0/4

CI = confidence interval; DEE = developmental and epileptic encephalopathies; EIMFS = epilepsy of infancy with migrating focal seizures; EMAS = epilepsy with myoclonic-atonic seizures; GDD = global developmental delay; GEFS+ = generalized epilepsy with febrile seizures plus; GOF = gain-of-function; ID = intellectual disability; LGS = Lennox–Gastaut syndrome; LOF = loss-of-function; MAE = myoclonic atonic epilepsy.

### Correlating patient severity with functional data

The striking difference in age of first seizure onset (0.03–15 months) between patients is particularly important, as it represents an objective numerical parameter relatively straightforward to assess, is comparable across different clinicians, and can be used as a predictor of clinical outcomes of a patient. Typically, the earlier the onset the more severe the clinical outcomes. To investigate whether the severity of the clinical phenotype (age of onset) correlates with the functional consequence of the variant (ΔlogEC_50_), we performed linear regression for 31 patients (age of seizure onset unknown for one patient) harbouring one of 20 unique gain-of-function *GABRB3* variants (Fig. 1A). Surprisingly, only a weak correlation was shown (R^2^ = 0.10) with outliers outside the 95% CI range in both directions. By colour coding variants according to their structural location in the subunit protein, a clear pattern emerged (Fig. 1B and C). Patients with variants that reside in the M1 transmembrane helix (M1) present below the line of best fit (lower age of onset), while patients with variants in the coupling regions present above (older age of onset). To investigate whether this correlates with differences in desensitization profiles, we analysed the desensitization characteristics of all known *GABRB3* gain-of-function variants and representative loss-of-function variants.

### Measuring desensitization properties at concatenated receptors

**Figure 2:**
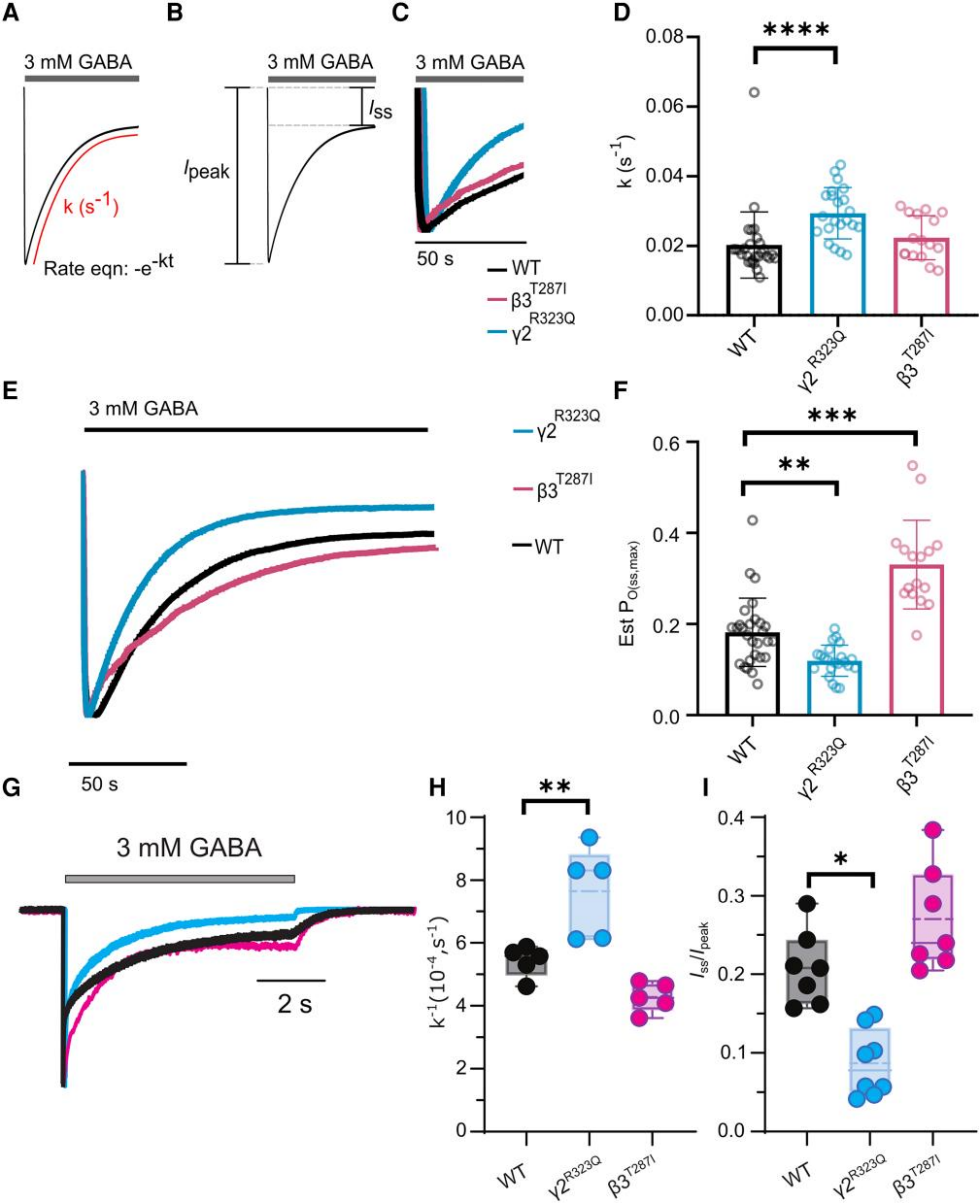
**Determination of key empirical parameters affected by desensitization**. (**A**) Current decay rates were determined by applying 3 mM GABA (grey bar) to oocytes expressing variant or wild-type (WT) concatenated receptors. Traces were fitted to an exponential decay (red line), −e^−kt^, to estimate the rate constant, k (s^−1^). (**B**) Steady-state currents were determined by subtracting the asymptote of the exponential decay from the holding current (*I*_ss_). (**C**) Representative traces of responses to 50 s application of 3 mM GABA at WT (black), γ2^R323Q^ (aqua) and β3^T287I^ (pink) variant receptors to illustrate current decay rates. (**D**) Bar graph of current decay rate constants. (**E**) Representative traces for determination of Est P_O(ss,max)_; 3 mM GABA was applied for 150 s and fitted to an exponential curve. The difference between the asymptote and holding current was transformed to the Est P_O(max)_. (**F**) Bar graph of the Est P_O(ss,max)_. (**G**) Representative traces for determination of desensitization time constants and steady-state currents at WT (black), γ2^R323Q^ (aqua) and β3^T287I^ (pink) variant receptors recorded from transfected HEK293 cells. (**H**) Box and whisker plot of desensitization current decay constant. (**I**) Box and whisker plot of steady-state currents. For all graphs, bars represent mean ± SD, circles represent individual experiments; *P < 0.05, **P < 0.01, ***P < 0.001 and ****P < 0.0001 compared to WT, non-parametric ANOVA with Dunn’s *post hoc* test. Est P_O(max)_ = maximum receptor open probability; Est P_O(ss,max)_ = maximum steady-state open probability; M1 = M1 transmembrane helix; M2 = M2 transmembrane helix.

Macroscopic desensitization properties of GABA_A_ receptors can be measured empirically in two main ways. First, the speed at which the current declines when the receptor binding sites are saturated with GABA, or the current decay rate [k (s^−1^)], describes the timeframe from the initial activation phase where maximum current flows through the ion channel until the current has declined to reach an equilibrium between open and desensitized states, a proxy for fast desensitization (Fig. 2A). Second, the steady-state open probability [P_O(ss,max)_], derived from the residual currents (*I*_ss_/*I*_peak_) describes the degree of residual current when this equilibrium is reached, a measure of desensitization at equilibrium where an increased P_O(ss,max)_ value corresponds to reduced desensitization at equilibrium (Fig. 2B).

In a series of recent articles, Gielen and colleagues have argued that two-electrode voltage clamp electrophysiology of concatenated receptors is well-suited to measuring receptor desensitization with substantially reduced variability in the kinetic parameters (reviewed in Gielen and Corringer^31^ and Gielen *et al*.^32^). The expression of concatenated α1β3γ2 receptors ensures a specific receptor population is at the cell surface, while expression of free subunits leads to cell-to-cell variability from mixed populations of α1β3 and α1β3γ2 receptors at the cell surface, but a potential caveat is that concatenation of the receptor may affect receptor activation or expression.^27^ To investigate this, we injected oocytes with the WT concatenated γ2-β3-α1-β3-α1 alone, α1 and β3 subunits in a 5:1 ratio, or α1, β3 and γ2 subunits in either a 5:1:5 or 10:1:10 ratio and measured the maximum currents, maximal estimated open probability, current decay rates, steady-state currents at equilibrium and GABA sensitivity. Although caution should be taken when using current amplitudes to assess channel surface expression, concatenation did not appear to hinder surface expression, with similar maximum currents between concatenated or free subunits expressed at a 10:1:10 ratio. Binary α1β3 receptors also strongly expressed at similar levels, while maximum currents were significantly increased at receptors injected with a 5:1:5 ratio that likely expresses a mixture of binary and ternary complexes (Supplementary Fig. 1 and Supplementary Tables 1 and 2).

To investigate if binary receptors would introduce variation to desensitization parameters, we measured steady-state currents at oocytes expressing α1β3 receptors alone compared to oocytes injected with RNA encoding concatenated α1β3γ2 receptors, or free α1, β3 and γ2 subunits. Steady-state currents, expressed either as *I*_ss_/*I*_peak_ or Est P_O(ss,max)_ were significantly larger at oocytes expressing binary α1β3 receptors than α1β3γ2 receptors expressed either by free subunits or concatenated constructs (Supplementary Fig. 1 and Supplementary Tables 1 and 2).

As binary α1β3 receptors are commonly expressed at appreciable levels from either injection into oocytes or transfection into mammalian cells of free α, β and γ subunits,^27,33^ the higher steady-state currents of binary receptors will increase variability whenever free subunits are used. Our results show, that tethered subunits did not significantly affect receptor activation properties or expression levels. Therefore, we used the concatenation expression system in *Xenopus* oocytes where a single variant β3 subunit is introduced into the pentameric receptor ensuring both that there is no contamination of the recordings from binary receptors, and that homogenous receptor populations of the most common receptor in patients heterozygous for the *GABRB3* variant are expressed. Further detailed evaluation and comparison of the concatenated and free constructs are found in the Supplementary material.

### Validating desensitization properties in different expression systems

To validate our experimental design of comparing concatenated receptor variants, we initially compared current decay rates and steady-state open probabilities for two previously described patient variants, γ2^R323Q^ and β3^T287I^, to WT. We then expressed these variants with DNA encoding α1, β3 and γ2 subunits in HEK293 cells to ensure the results were consistent in both *Xenopus* and mammalian expression systems. A concentration of 3 mM GABA was chosen as this is a reasonable mimic of the estimated peak concentrations of GABA at the synaptic cleft.^34^ The γ2^R323Q^ variant was previously reported to accelerate current decay rates at high GABA concentrations, whereas no effects were noted for the β3^T287I^ variant.^11,16^ To investigate this, an exponential decay function was fitted to 150-s long responses of 3 mM GABA (Fig. 2C, Table 2 and Supplementary Fig. 2). The current decay constant was significantly increased by the γ2^R323Q^ but not the β3^T287I^ variant, indicating faster desensitization rates at the γ2^R323Q^ variant (Fig. 2D and Table 2). Evaluating steady-state currents requires knowledge of the degree by which receptors respond to 3 mM GABA applications [i.e. estimated maximal open probability, Est P_O(max)_]. The Est P_O(max)_ was reduced for the γ2^R323Q^, but unchanged for the β3^T287I^ variant (Table 2 and Supplementary Tables 3 and 4). To determine the steady-state open probability [Est P_O(ss,max)_], an exponential decay function was fitted to 150-s-long 3 mM GABA responses to determine the steady state currents relative to the peak (*I*_ss_/*I*_peak_), and the asymptote normalized to the Est P_O(max)_ value (Fig. 2E and Supplementary Tables 3 and 4). Compared to WT, the desensitization at equilibrium was significantly increased at γ2^R323Q^ but decreased for β3^T287I^ (Fig. 2F and Table 2).

**Table 2:** Desensitization parameters of gain-of-function *GABRB3* variants

Variant^a^	ΔLogEC_50_^b^	k	*P*-value^c^	Est P_O(ss,max)_	*P*-value^c^	Est P_O(max)_	*P*-value^c^	*n* ^d^
**Group 1^e^**								
Wild-type	–	0.02 ± 0.0095	–	0.18 ± 0.075	–	0.98 ± 0.22	–	28
γ2R323Q	–	0.029 ± 0.0074	<0.0001	0.12 ± 0.034	0.008	0.77 ± 0.2	0.0047	22
T287I	0.52	0.022 ± 0.0063	0.2232	0.33 ± 0.097	0.0004	1.1 ± 0.18	0.0988	16
**Group 2** ^f^								
Wild-type	–	0.027 ± 0.009	–	0.11 ± 0.034	–	0.8 ± 0.15	–	32
T281A	0.90	0.038 ± 0.0091	n.d.*	0.18 ± 0.036	0.025	1 ± 0.26	0.1054	12
L284M	0.67	0.033 ± 0.0075	n.d.*	0.1 ± 0.035	>0.9999	0.81 ± 0.16	>0.9999	13
L284P	1.22	0.034 ± 0.021	n.d.*	0.33 ± 0.11	<0.0001	0.89 ± 0.28	0.5601	14
L284R	1.09	0.03 ± 0.011	n.d.*	0.5 ± 0.19	<0.0001	1.2 ± 0.60	0.0159	15
T288N	0.45	0.03 ± 0.0076	n.d.*	0.096 ± 0.032	>0.9999	0.94 ± 0.22	0.2654	13
L293H	0.50	0.033 ± 0.009	n.d.*	0.078 ± 0.023	0.303	0.84 ± 0.3	>0.9999	15
**Group 3** ^g^								
Wild-type	–	0.025 ± 0.0061	–	0.10 ± 0.022	–	0.85 ± 0.11	–	19
E77K	0.64	0.049 ± 0.011	<0.0001	0.098 ± 0.036	>0.9999	0.81 ± 0.15	n.d.*	12
V78F	0.40	0.036 ± 0.0065	0.023	0.086 ± 0.071	0.241	0.81 ± 0.19	n.d.*	15
L170R	0.26	0.029 ± 0.0082	>0.9999	0.1 ± 0.034	>0.9999	0.85 ± 0.1	n.d.*	15
A305T	0.46	0.046 ± 0.0088	<0.0001	0.12 ± 0.028	>0.9999	0.94 ± 0.14	n.d.*	11
A305V	0.53	0.044 ± 0.0069	<0.0001	0.13 ± 0.043	0.823	0.88 ± 0.11	n.d.*	11
I306T	0.38	0.034 ± 0.0093	0.067	0.11 ± 0.036	>0.9999	0.79 ± 0.09	n.d.*	14
**Group 4** ^h^								
Wild-type	–	0.029 ± 0.0052	–	0.079 ± 0.021	–	0.74 ± 0.11	–	13
L124F	0.50	0.039 ± 0.0078	0.051	0.084 ± 0.049	>0.9999	0.83 ± 0.21	n.d.*	12
T185I	0.59	0.036 ± 0.0046	0.313	0.081 ± 0.043	>0.9999	0.9 ± 0.26	n.d.*	14
Y245H	0.37	0.02 ± 0.0048	0.573	0.15 ± 0.04	0.016	0.79 ± 0.16	n.d.*	12
S254F	0.24	0.05 ± 0.027	0.005	0.19 ± 0.065	0.002	0.87 ± 0.1	n.d.*	11
L256Q	0.47	0.034 ± 0.0062	>0.9999	0.17 ± 0.088	0.014	0.89 ± 0.16	n.d.*	12
I280F	0.65	0.058 ± 0.0012	<0.0001	0.076 ± 0.052	>0.9999	0.86 ± 0.18	n.d.*	17
**Group 5** ^i^								
Wild-type	–	0.028 ± 0.0094	–	0.15 ± 0.038	–	0.99 ± 0.26	–	24
I300T	0.99	0.036 ± 0.0096	0.121	0.048 ± 0.061	<0.0001	0.92 ± 0.32	>0.9999	20

Errors are given as standard deviations. Est P_O(max)_ = maximum receptor open probability; Est P_O(ss,max)_ = maximum steady-state open probability; k = rate constant of current decay.

^a^Unless otherwise specified, variants are in the β3 subunit.

^b^Values taken from Absalom *et al*.^9^

^c^One-way Kruskal–Wallis ANOVA followed by a Dunn’s *post hoc* test, experimental groups compared separately.

^d^Numbers of individual oocytes to determine k, Est P_O(max)_ and Est P_O(ss,max)_.

^e-i^Experimental groups where variants and wild-type performed on same day.

^*^n.d. = not determined, as *P* > 0.05 for initial ANOVA.

These data show that the γ2^R323Q^ variant increases desensitization both through accelerated current decay rates and increased desensitization at equilibrium, both phenomena that reduce total charge transfer upon receptor activation (decreased area under curve). In contrast, the β3^T287I^ variant reduced desensitization at equilibrium with a greater percentage of receptors remaining open during a prolonged application of GABA, allowing net greater flow of chloride through the β3^T287I^ receptors compared to WT (increased area under curve). These results demonstrate that desensitization properties can be significantly altered in either direction by pathogenic variants.

Recent kinetic modelling has proposed that variants in the β2 subunit of α1β2γ2 receptors modulate the slow component of GABA_A_ receptor desensitization.^31^ Therefore, we fitted the decay currents to a two-phase exponential function for the WT and β3^T287I^ receptors. Although there was a significant increase in the fraction of the fast component at β3^T287I^ receptors, we did not identify any changes in the weighted time constant (τ_weighted_) or slow time component (τ_slow_) (Supplementary Tables 5 and 6). Decay currents at γ2^R323Q^ receptors did not fit to two-phase exponential functions and were not analysed by this method.

To confirm that the results we measure in a concatenated receptor construct expressed in a *Xenopus* oocyte are equivalent to receptors expressed in a mammalian system, HEK293 AD cell expression was used to investigate the kinetic properties of WT α1β3γ2 and receptors containing γ2^R323Q^ and β3^T287I^ variants. Relatively long applications of 3 mM GABA to small cells or excised membrane patches produced desensitizing currents that were adequately fit to two exponential equations for all three receptor types (Fig. 2G and Supplementary Tables 7 and 8). Plotting the weighted desensitization rate constant (k^−1^) demonstrated that γ2^R323Q^-containing receptors decayed more rapidly than WT, whereas the decay rate for the β3^T287I^-containing receptors was similar to that of WT (Fig. 2H). The remaining steady-state current (*I*_ss_) at the end of the GABA application was also measured and normalized to the peak current (*I*_ss_/*I*_peak_). The plots of this data show that the γ2^R323Q^ variant results in a reduced steady-state current, whereas the β3^T287I^ variant steady-state current was not significantly increased compared to WT receptors (Fig. 2I). Detailed measurements of simulated synaptic currents are found in the Supplementary material.

Overall, the kinetic parameters of the two variant receptors are consistent with a ‘loss-of-function’ for α1β3γ2^R323Q^ receptors and a ‘gain-of-function’ for the α1β3^T287I^γ2 receptors, and thus broadly similar conclusions can be drawn for variants expressed in concatenated constructs in *Xenopus* oocytes or as free subunits in mammalian cells.

### *GABRB3* M2 and M1 variants

**Figure 3:**
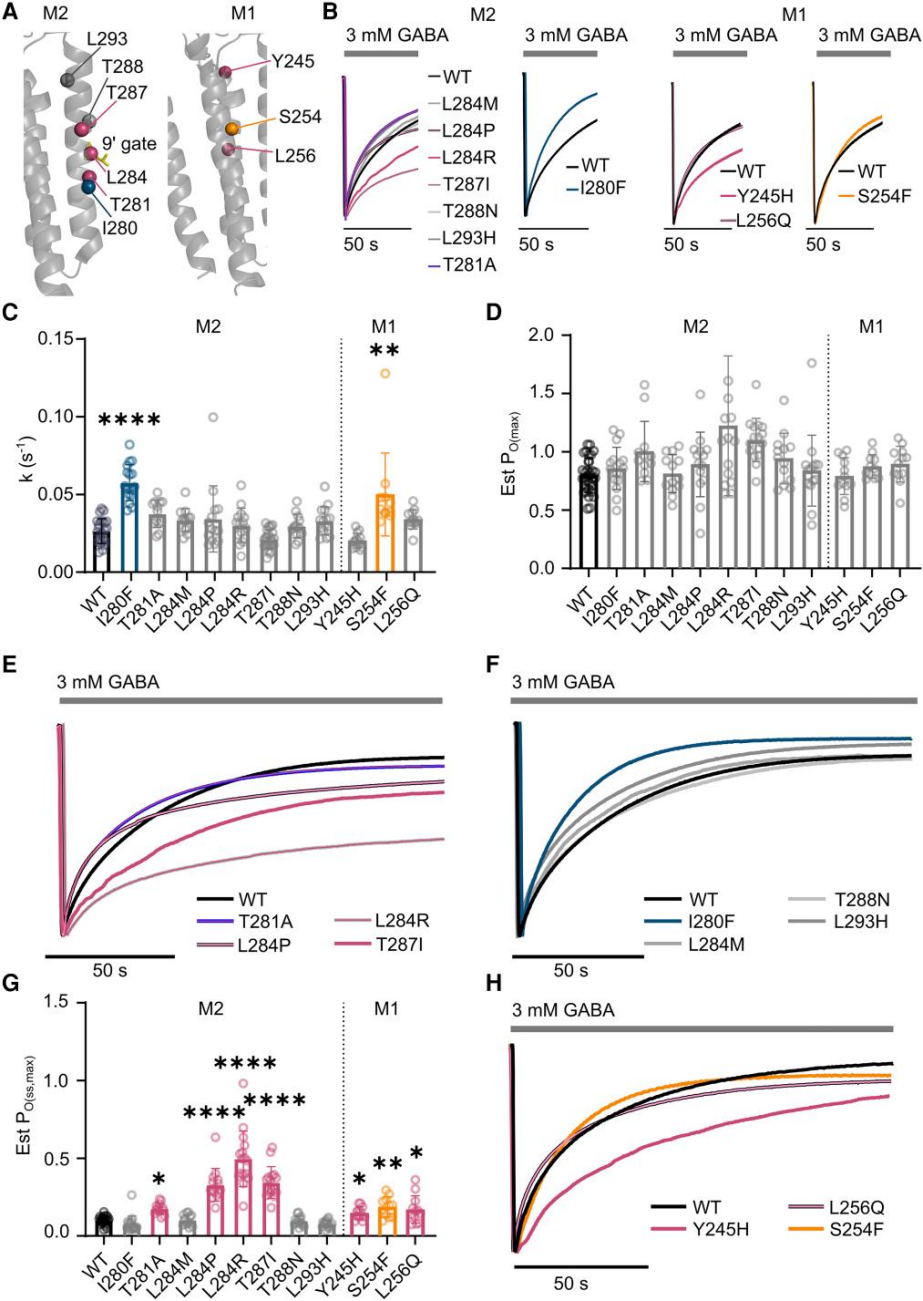
**Determination of key empirical parameters related to desensitization of variants at the transmembrane regions**. (**A**) Location of M2 (*left*) and M1 (*right*) variants (depicted as spheres) in the cryogenic electron microscopy structure of the GABA_A_ receptor β3 subunit, yellow indicates the 9′ gates (PDB:6HUP). Orange denotes significantly faster decay constants and higher Est P_O(ss,max)_, blue denotes only significantly faster decay constants, pink denotes only significantly higher Est P_O(ss,max)_ and grey denotes no change. (**B**) Representative traces for M2 variants with unchanged current decay rates at (*left*) wild-type (WT), β3^L284M^, β3^L284R^, β3^L284P^ (black border), β3^T287I^ (grey border), β3^T281A^ (blue border), β3^T288N^ (light grey) and β3^L293H^ (dark grey) receptors, and faster decay rates at (*right*) WT (black) and β3^I280F^ receptors. Representative traces for M1 variants unchanged current decay rates at (*left*) WT, β3^Y245H^, and β3^L256Q^ (black border) receptors, and fast current decay rates at WT (black) and β3^S254F^ receptors. (**C**) Bar graph of current decay rates. (**D**) Bar graph of Est P_O(max)_. (**E**) Representative traces of 150 s GABA application at M2 variants with increased Est P_O(ss,max)_ at WT, β3^L284P^, β3^L284R^, β3^T281A^ and β3^T287I^ variants. (**F**) Representative traces for M2 variants with unchanged Est P_O(ss,max)_ at WT, β3^I280F^, β3^L284M^, β3^T288N^ and β3^L293H^ receptors. (**G**) Bar graph of Est P_O(ss,max)_. (**H**) Representative traces of 150 s GABA application at M1 variants with increased Est P_O(ss,max)_. For all bar graphs, bars represent mean ± SD, circles represent individual experiments; *P < 0.05, **P < 0.01, ***P < 0.001 and ****P < 0.0001 compared to WT, non-parametric ANOVA with Dunn’s *post hoc* test. Coloured bars and circles represent significant differences compared to WT, grey bars and circles are not significant. Est P_O(max)_ = maximum receptor open probability; Est P_O(ss,max)_ = maximum steady-state open probability; k = rate constant of current decay; M1 = M1 transmembrane helix; M2 = M2 transmembrane helix.

In GABA_A_ receptors, the M2 helix lines the channel pore and variants in this region are associated with greater clinical severity (Fig. 3A).^3,4,8,13,14^ In our cohort, there is a cluster of eight gain-of-function epilepsy-associated variants in the M2 region, including three separate variants at the central leucine residue, commonly referred to as the central 9′-leucine residue, that forms the hydrophobic channel gate.^35^ No significant difference in current decay rates was observed for seven M2 helix variants, including the β3^T287I^ (Fig. 3B), and only the β3^I280F^ variant significantly accelerated the current decay rate compared to WT (Fig. 3C and Table 2). There was no difference in Est P_O(max)_ between variants (Table 2 and Fig. 3D). Desensitization at equilibrium were significantly decreased for four variants, two located at the central leucine residue compared to the WT (β3^L284P^ and β3^L284R^) as well as β3^T281A^ and β3^T287I^ (Fig. 3E and F). The remaining variants did not significantly change the Est P_O(ss,max)_ (Fig. 3G and Table 2), and only the β3^L284P^ had reduced maximum current amplitudes (Supplementary Fig. 3 and Supplementary Tables 3 and 4).

The second structural region associated with more severe variants is the M1 region (Fig. 3A).^20^ Our analysis of M1 variants revealed that the β3^S254F^ variant significantly accelerated current decay rates, whereas no changes were observed for β3^Y245H^ and β3^L256Q^ (Fig. 3B and C and Table 2). There was also no difference in Est P_O(max)_ (Fig. 3D and Table 2). However, the desensitization at equilibrium was reduced at all three M1 variants (Fig. 3G and H and Table 2).

We then fitted the decay currents to a two-phase exponential decay current. The weighted time constant was significantly accelerated at the β3^I280F^ and decelerated at the β3^Y245H^ receptors. The slow time component was also accelerated at the β3^I280F^ and decelerated at the β3^Y245H^ and β3^L284P^ receptors, while the fraction of the fast component was increased at the β3^L256Q^, β3^L284R^ and β3^L284P^ receptors (Supplementary Tables 5 and 6). This is consistent with previous reports of the importance of the β3 subunit in the slow time component of current decay.^31^

In summary, the current decay rate of the receptor is appreciably accelerated by one variant in the M2 region (β3^I280F^) and one in the M1 region (β3^S254F^). During synaptic transmission, this increase in current decay may reduce the current passing across the cell membrane. Nonetheless, all variants in the M1 region and four out of eight variants in the M2 region (β3^T281A^, β3^L284P^, β3^L284R^ and β3^T287I^) significantly reduced desensitization at equilibrium. In cases of prolonged periods of activation, this would be expected to increase the pool of receptors available for reactivation and thus increase the inhibitory currents flowing across the cell membrane. This indicates that these variants have enhanced gain-of-function compared to those that have no change in current decay rates.

### *GABRB3* coupling region and extracellular variants

**Figure 4:**
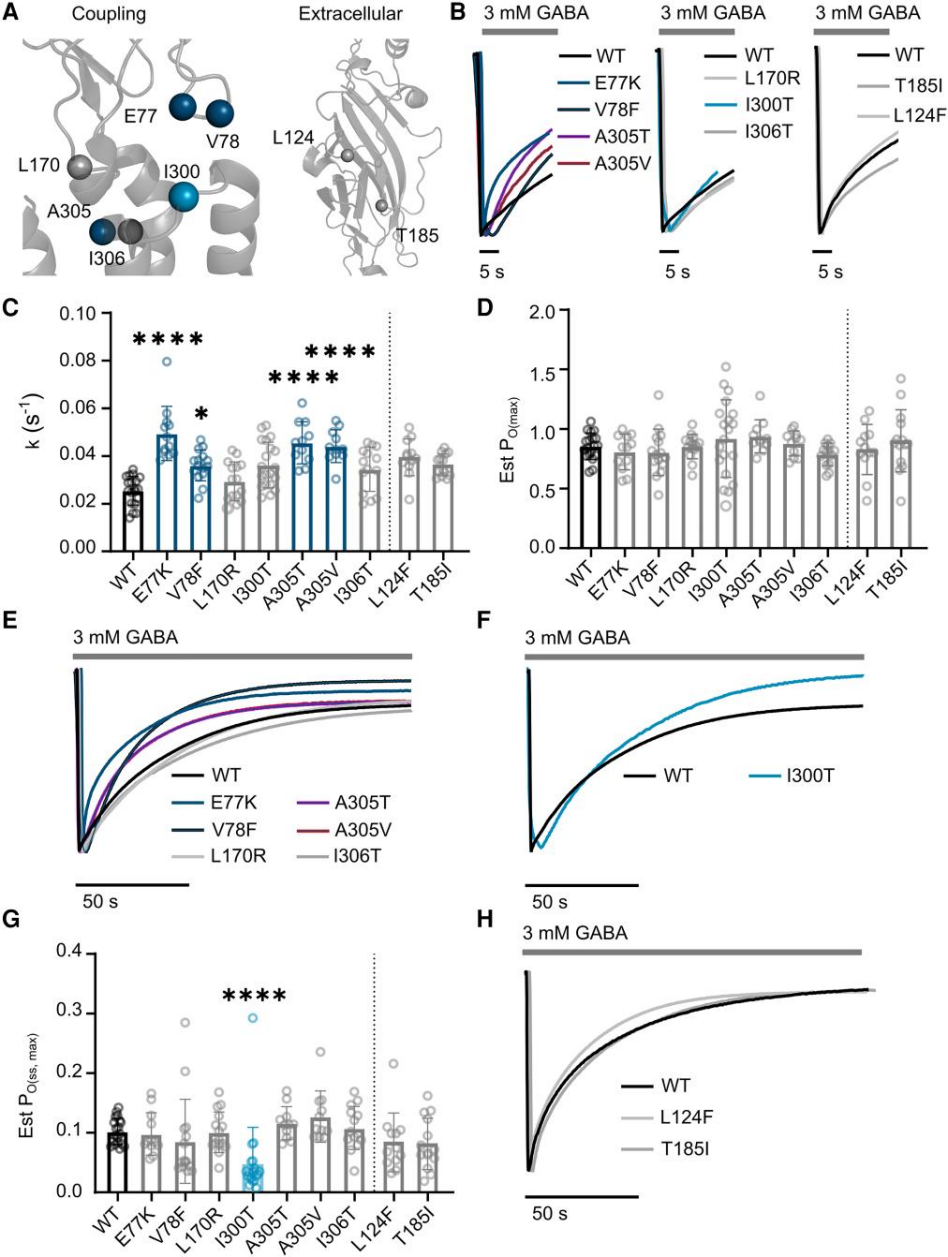
**Determination of key empirical parameters related to desensitization of variants at the coupling and extracellular regions**. (**A**) Location of coupling loop (*left*) and extracellular (*right*) variants (spheres) within the cryogenic electron microscopy structure of β3 subunit of the GABA_A_ receptor (PDB:6HUP). Blue denotes only significantly faster decay constants, aqua denotes only significantly lower Est P_O(ss,max)_ and grey denotes no change. (**B**) Representative traces for coupling loop variants with faster current decay rates (*left*) at wild-type (WT), β3^E77K^, β3^V78F^ (black border), β3^A305T^ (purple border) and β3^A305V^ (red border) receptors and unchanged decay rates (*middle*) at WT (black), β3^L170R^ (light grey), β3^I300T^ (aqua) and β3^I306T^ (grey) receptors. Representative traces for extracellular variants with no changes in decay rates (*right*) at WT, β3^L124F^ and β3^T185I^ receptors. (**C**) Bar graph of current decay rates. (**D**) Bar graph of Est P_O(max)_. (**E**) Representative traces of 150 s GABA application for unchanged Est P_O(ss,max)_ at WT and coupling variants. (**F**) Representative trace of 150 s GABA application for reduced Est P_O(ss,max)_ variant, β3^I300T^ (**G**) Bar graph of Est P_O(ss,max)_. In all bar graphs, bars represent mean ± SD, circles represent individual experiments; *P < 0.05, **P < 0.01, ***P < 0.001 and ****P < 0.0001 compared to WT, non-parametric ANOVA with Dunn’s *post hoc* test. Coloured bars and circles represent significant differences compared to WT, grey bars and circles are not significant. (**H**) Representative traces of 150 s GABA application for unchanged Est P_O(ss,max)_ at WT and extracellular region variants. Est P_O(max)_ = maximum receptor open probability; Est P_O(ss,max)_ = maximum steady-state open probability; k = rate constant of current decay.

The coupling region linking the extracellular and transmembrane regions is enriched with pathogenic variants.^9^ Seven gain-of-function variants have been identified in the coupling region comprising of the extracellular β1–2, β6–7 and the transmembrane M2–M3 loops that alter conformation in the transitions between closed, intermediate and open states (Fig. 4A). Current decay rates were significantly accelerated by the β3^E77K^ and β3^V78F^ variants located in the β1–2 loop and the β3^A305T^ the β3^A305V^ variants located in the M2–M3 coupling loop, while the rates of the β3^L170R^, β3^I300T^ and β3^I306T^ variants were not significantly altered (Fig. 4B and C and Table 2). There was no difference in Est P_O(max)_ for all variants (Fig. 4D). For six of the seven variants, no changes to the desensitization at equilibrium were observed. However, the desensitization at equilibrium was increased at the β3^I300T^ receptor (Table 2 and Fig. 4E–G). Only the β3^V78F^ and β3^I300T^ had reduced maximum current amplitudes, that may be a result of reduced surface expression or a consequence of decreased activation times (Supplementary Fig. 3 and Supplementary Tables 3 and 4). We then fitted the decay currents to a two-phase exponential decay current. Both the weighted time constant and the slow time component was significantly accelerated at the β3^E77K^, β3^I300T^, β3^A305T^ and β3^A305V^ receptors, in agreement with previous reports that the β3 subunit coordinates the slow time component of current decay (Supplementary Tables 5 and 6).^31^

Finally, the extracellular region apart from the coupling region contains two gain-of-function variants, the β3^L124F^ and β3^T185I^. There were no changes seen for the β3^L124F^ and β3^T185I^ variants in current decay rates, Est P_O(max)_ or steady-state activity, and thus did not alter either aspect of desensitization (Fig. 4). No parameters were significantly changed when current decay was fitted to a two-phase exponential function, and neither variant had a change in maximum current amplitudes (Supplementary Fig. 3 and a Supplementary Tables 3 and 4).

Therefore, most gain-of-function variants in the coupling region accelerate the current decay rate of receptors at high concentrations of GABA, but do not affect the steady-state currents. Variants in the extracellular domain outside of the coupling region did not affect either aspect of desensitization.

### *GABRB3* loss-of-function variants

**Figure 5:**
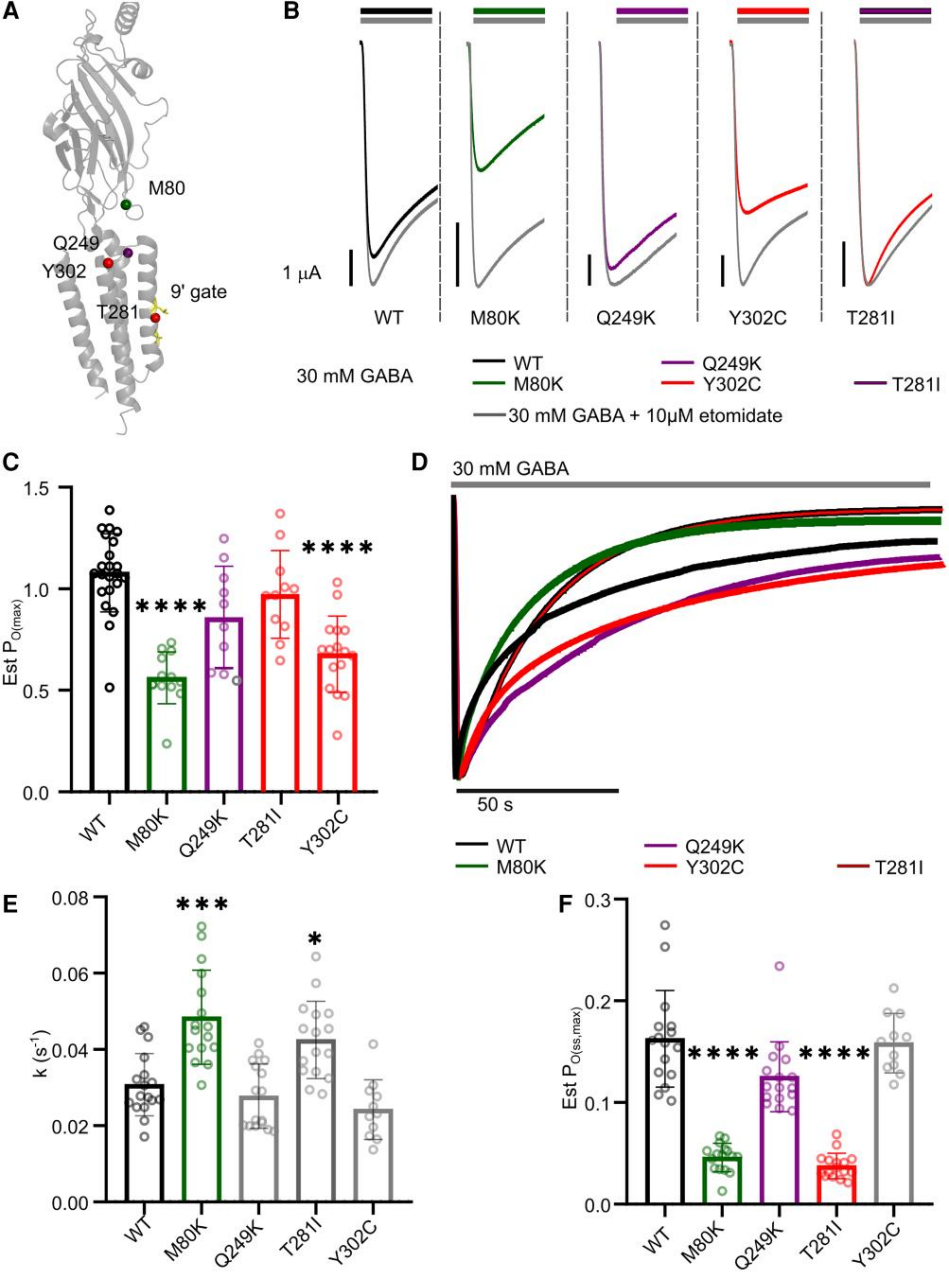
**Determination of key empirical parameters affected by desensitization of loss-of-function variants**. (**A**) Location of loss-of-function variants (spheres) within the cryogenic electron microscopy structure of β3 subunit of the GABA_A_ receptor (PDB:6HUP), 9′ gate of the M2 transmembrane helix highlighted in yellow. Green denotes significantly faster decay constants, decreased Est P_O(max)_ and Est P_O(ss,max)_, purple denotes significantly decreased Est P_O(max)_ and Est P_O(ss,max)_, red denotes only decreased Est P_O(max)_ and grey denotes no change. (**B**) Representative trace of currents elicited by 30 mM GABA overlayed with trace elicited by 30 mM GABA + 10 μM etomidate (grey) for β3^M80K^, β3^Q249K^, β3^T281I^ and β3^Y302C^. (**C**) Bar graph of Est P_O(max)._ (**D**) Representative traces of GABA application for current decay and Est P_O(ss,max)_ at wild-type (WT) and loss-of-function variants. (**E**) Bar graph of current decay rate at 30 mM GABA. (**F**) Bar graph of Est P_O(ss,max)_ determined with 30 mM GABA. For all graphs, bars represent mean ± SD, circles represent individual experiments; *P < 0.05, **P < 0.01, ***P < 0.001 and ****P < 0.0001 compared to WT, non-parametric ANOVA with Dunn’s *post hoc* test. Coloured bars and circles represent significant differences compared to WT, grey bars and circles are not significant. Est P_O(max)_ = maximum receptor open probability; Est P_O(ss,max)_ = maximum steady-state open probability; k = rate constant of current decay.

For comparison, we selected four loss-of-function variants from the same structural regions and evaluated changes in current decay and the desensitization equilibrium. These variants included β3^M80K^ and β3^Y302C^ in the β1–2 and M2-M3 coupling loops respectively, β3^Q249K^ in the M1 and β3^T281I^ in the M2 region (Fig. 5A). After application of 30 mM GABA, the β3^M80K^ had a significant reduction in the maximal current amplitudes, and both the β3^M80K^ and β3^Y302C^ receptors had a significant reduction in P_O(max)_, which may reflect the reduced gating efficiency (Fig. 5B and C and Supplementary Table 9). The β3^M80K^ and β3^T281I^ receptors had significantly accelerated current decay rates and reduced desensitization at equilibrium, while neither the current decay rates or desensitization at equilibrium was significantly changed at the β3^Q249K^ and β3^Y302C^ receptors (Fig. 5D–F and Supplementary Tables 4 and 9). Taken together, loss-of-function variants displayed either unchanged or increased desensitization properties, either by accelerated current decay rates or increasing desensitization at equilibrium, indicating that there’s a further reduction in inhibitory activity of the receptor.

### Associations of desensitization characteristics with age of seizure onset

**Figure 6:**
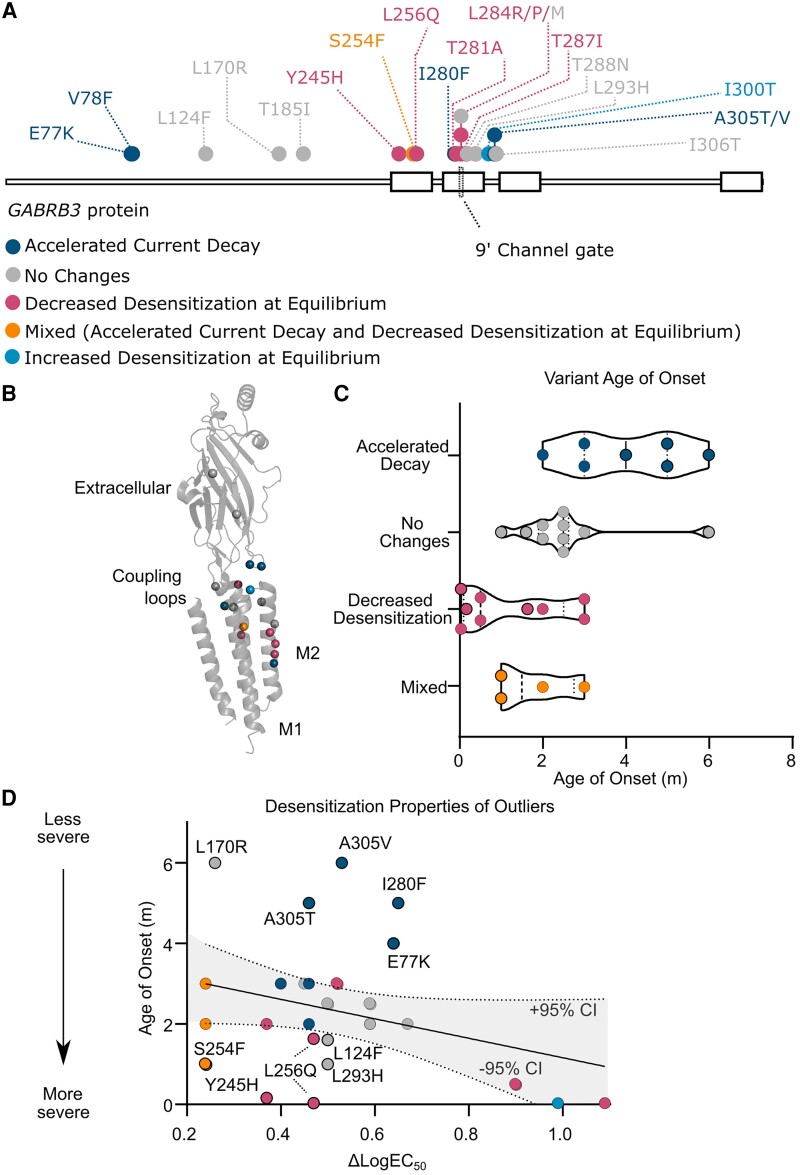
**Age of onset is affected by desensitization at gain-of-function variants**. (**A**) 2D representation of the protein sequence of the β3 subunit of the GABA_A_ receptor. Gain-of-function variants are represented as dots, with colours representing different variant desensitization properties of (i) accelerated current decay (blue); (ii) no change in desensitization (grey); (iii) reduced desensitization at equilibrium (pink); (iv) mixed with accelerated current decay and reduced desensitization at equilibrium (orange); and (v) increased desensitization at equilibrium (light blue). (**B**) 3D representation of a single β3 subunit of the GABA_A_ receptor, with the location of variants shown as spheres and colour coded in the same manner. (**C**) Truncated violin plot comparing age of seizure onset between variants with different changes in desensitization. Colours represent accelerated current decay (blue), no change in desensitization (grey), reduced desensitization at equilibrium (pink), and mixed with accelerated current decay and reduced desensitization at equilibrium (orange). Black outline denotes variants are outliers in the linear regression of age of onset and ΔlogEC_50_. (**D**) Scatter plot of patient age of seizure onset and the extent of change in logEC_50_ comparing variants. The line of best fit for a linear regression of all variants is shown and the area within the 95% confidence intervals is shaded in grey. m = months.

Mapping variants to common structural motifs showed consistent patterns in changes to desensitization properties (Fig. 6A and B). Variants in the coupling loops were more likely to display accelerated current decay, whereas the M1 and M2 regions more likely to increase steady-state currents. To determine if these differences in desensitization properties affected the severity of the phenotype, clinical data for the 32 patients (31 with known age of seizure onset) with gain-of-function variants was segregated into four categories: (i) No changes to desensitization; (ii) Accelerated current decay; (iii) Decreased desensitization at equilibrium; and (iv) Mixed effects of accelerated current decay and decreased desensitization at equilibrium (Table 1).

Patients for which no desensitization changes were observed had an age of seizure onset of 2.5 months (Fig. 6C and Table 1). Patients with variants that accelerated current decay rates had an older age of seizure onset of 4 months, whereas patients with decreased desensitization at equilibrium had a lower age of seizure onset of 0.5 months. Patients with mixed desensitization properties resembled those with only decreased desensitization at equilibrium with an early onset of 1.5 months.

While these data suggest that accelerated current decay and increased steady-state currents influence the age of seizure onset in opposite directions, this analysis ignores the magnitude of change in GABA sensitivity, where a younger age of onset is generally associated with very large ΔlogEC_50_ changes. Therefore, desensitization properties were incorporated by colour coding variants in our correlation between age of onset and ΔlogEC_50_ (Fig. 6D). This revealed that data above and below the line of best fit coincided with changes in desensitization properties. Variants above the line (older age of onset) displayed accelerated current decay, and variants below the line (younger age of onset) decreased desensitization at equilibrium. Variants with both an accelerated current decay and decreased desensitization at equilibrium either were below or on the line. Hence, at gain-of-function variants, accounting for changes in desensitization properties helps in clarifying the poor correlation between the functional change of the variant and the age of seizure onset.

### Correlation of other clinical features with receptor desensitization

Other observations emerged when comparing the clinical features among the groups of patients with gain-of-function variants. In particular, the group with accelerated current decay differed from the three remaining groups. For the eight patients harbouring variants with accelerated current decay, there were no reports of early mortality, movement disorders or EIMFS (all 0/8), compared to reported total instances of early mortality (5/24), movement disorders including dystonia and dyskinesia (10/24) and EIMFS (9/24) for the other groups collectively. No differences were observed for other clinical features of microcephaly (3/8 and 10/24) and severe intellectual disability (3/8 and 16/24), with similar occurrences seen in all groups.

## Discussion

Understanding of the mechanisms of disease is highly important for accurate diagnosis and pharmacological intervention in DEE. Here, we aim to unravel the reason behind a strong association between the structural location of *GABRB3* variants and the severity of the clinical phenotype even within a cohort consisting of only gain-of-function patients. Although altered desensitization properties have been proposed by several groups to influence the net effect of variants in DEE,^16-19,36^ no direct evidence for such an association has been provided. Therefore, we evaluated the desensitizing properties of 20 gain-of-function and four loss-of-function *GABRB3* variants and correlated the data with the age of onset of patients harbouring these variants. We provide the first evidence that changes in desensitization properties are linked to the structural location within the protein and identify correlations with the clinical outcomes of patients.

### Variants can either increase or decrease receptor desensitization

The desensitization properties of GABA_A_ receptor variants have not previously been studied in a systematic manner, and often focused solely on current decay rates.^16-19^ Our data reveals more intricate effects of variants on receptor desensitization. The loss-of-function variants presented were relatively straightforward as they either did not change or increased desensitization properties. Variants that increased desensitization would be expected to further reduce GABAergic currents already impaired by the loss in GABA sensitivity and thereby exacerbate the loss-of-function characteristics. By contrast, the gain-of-function variants presented in a complex manner and could be grouped into four different categories based on their desensitization characteristics: (i) no change to desensitization; (ii) accelerated current decay rates (i.e. increased desensitization); (iii) reduced desensitization at equilibrium; and (iv) accelerated current decay and reduced desensitization at equilibrium (i.e. mixed desensitization effects). Generally, variants with accelerated current decay were more likely to be located in the coupling region, and variants with reduced desensitization at equilibrium were more likely to be located in the transmembrane regions.

### Why are variants in transmembrane regions associated with worse clinical outcomes?

As more GABA_A_ receptor variants are identified, a clear separation has occurred in the severity of clinical outcomes between patients harbouring variants in the M1 and M2 transmembrane domains and patients with variants located elsewhere.^3,4,8,13,14^ For *GABRB3* variants, one reason for this is that the overall clinical presentation is strongly associated with changes in the GABA sensitivity, and transmembrane regions have a greater proportion of gain-of-function variants.

However, given the remarkable consequences of gain- or loss-of-function variants, we would predict that the magnitude of change of GABA sensitivity would correlate directly with disease severity. Although there is a trend for an earlier age of onset for variants with greater increases in GABA sensitivity, the overall correlation is surprisingly weak. Coupling region variants with no change in desensitization (β3^L170R^) or accelerated current decay (β3^A305T^, β3^A305V^ and β3^E77K^) presented above the line of best fit, while M1 variants with decreased desensitization at equilibrium (β3^Y245H^ and β3^L256Q^) or mixed desensitization effects (β3^S254F^) presented below the line. Indeed, while M1 gain-of-function variants increase GABA sensitivity by a relatively modest 0.24–0.47 ΔlogEC_50_ value, these patients have severe outcomes with a median age of onset of just 1.3 months, movement disorders such as dystonia and dyskinesia (3/8) and the rare and devastating DEE syndrome, EIMFS (3/8). Despite similar magnitudes of changes in GABA sensitivity, patients harbouring variants in the extracellular and coupling regions have an older median age of onset of 4 months. Hence, we propose that the conundrum of why transmembrane variants present with more severe clinical outcomes is 2-fold, they are more likely to cause gain-of-function and they are more likely to have decreased desensitization at equilibrium that further increase GABAergic currents.

### GABA sensitivity or desensitization—which determines the clinical outcome?

Previous studies have identified GABA_A_ receptor variants with counteracting biophysical properties of increased GABA sensitivity and reduced activity from accelerated current decay.^16-19,36^ In these cases, authors have, to greater or lesser extents, attributed clinical phenotypes to enhanced desensitization (loss-of-function) consistent with the classical paradigm that decreased GABAergic activity leads to epilepsy. However, such attributions were speculative and fundamentally excluded a significant part of the data. In our studies, we likewise observed variants with mixed biophysical properties (e.g. coupling region variants and β3^I280F^). This raises the question of which parameter determines the overall clinical outcome of the patient—increased GABA sensitivity or accelerated current decay?

Ultimately, the clinical phenotype is the logical arbiter of whether a variant is a gain- or loss-of-function. The genotype/phenotype correlations show patients harbouring gain-of-function variants with increased desensitization properties compared to those with loss-of-function variants are overall different. They have earlier seizure onset (median 4 months versus 10 months for loss-of-function), higher rates of severe or profound ID (7/8 versus 8/38) and higher rates of microcephaly (3/8 versus 0/38) (Table 1). Patients harbouring variants with no changes, mixed or decreased desensitization properties displayed the most severe clinical indications including EIMFS, movement disorders including dystonia and dyskinesia, and higher risk of early death (Table 1), and all patients harbouring gain-of-function variants shared the same characteristics of early age of onset, severe or profound ID and microcephaly. Overall, the clinical outcomes for patients harbouring variants with accelerated current decay resemble those for gain-of-function variants, albeit with milder symptoms for some indications.

Overall, the clinical outcomes for patients harbouring a gain- or loss-of-function variant provides strong evidence that GABA sensitivity is the primary determinant of the phenotype. A gain-of-function variant is thus not rendered a loss-of-function by accelerated current decay rates, and most likely a loss-of-function variant would not become a gain-of-function either if displaying increased steady state currents. The β3^I300T^ variant is an excellent example underscoring this. This variant decreased the steady-state open probability (loss-of-function) but has one of the largest changes in GABA sensitivity and youngest ages of onset. In this case, the profound shift in the GABA sensitivity appears to render changes in desensitization redundant. Accelerated current decay also appears not to protect against the effects of increased steady-state currents, as the β3^S254F^ variant with mixed desensitization properties overall resembled those with only increased steady state. Finally, some caution must also be taken when extrapolating the effects of increased desensitization from the receptor biophysical level to the increasing complexity of a neuron, a neuronal network and then neuronal development. For instance, the effect of increased desensitization properties on synaptic plasticity can be counterintuitive whereby recent experiments in dissociated rat hippocampal neurons showed that long term potentiation of inhibitory currents was increased by variants with increased desensitization properties.^37^

Hence, we propose that functional analysis of *GABRB3* variants should be interpreted primarily on changes to GABA sensitivity, and a combination of the structural location of the variant and desensitization analysis may assist in interpreting the severity of the variant. Other characteristics such as haploinsufficiency may also moderate the phenotype, however, this could not be evaluated here as we found no changes to the maximum current amplitudes for the gain-of-function *GABRB3* variants. To answer whether altered desensitization characteristics alone is sufficient to produce an epileptic phenotype requires a further discovery of such a variant, since all the ones tested here have altered GABA sensitivity.

### Cellular mechanisms and implications for treatment

The near ubiquitous spatial distribution of the β3 subunit in the brain and incorporation into subtypes mediating both phasic and tonic GABA currents complicates both the interpretation of disease mechanisms, and the expected outcomes of treatment.^38^ Their role at GABAergic inhibitory interneuron synapses is critical at loss-of-function variants where miniature inhibitory postsynaptic currents (mIPSCs) amplitudes are reduced and decay times slowed at cortical layer V/VI pyramidal neurons, mirroring loss-of-function *SCN1A* pathways where synaptic GABA is less frequently released.^39^

Gain-of-function variants, however, may disrupt different neuronal networks and cellular mechanisms depending on the magnitude of the functional changes. Increased tonic currents are likely to explain part of the phenotype, as gain-of-function *GARBD* variants have recently been reported with intellectual disabilities, but not movement disorders or EIMFS.^40^ Indeed, patients with *SLC6A1* loss of function variants, where impaired GABA transport function also would be predicted to increase tonic currents, also present with intellectual disabilities.^41^ However, movement disorders including dystonia, dyskinesia and choreoathetosis have been reported at *GABRB2* variants, including in the M1, M2 and M2-M3 regions, although it is not clear if these are gain- or loss-of-function variants,^8^ suggesting that the *GABRB2* and *GABRB3* may be active in similar motor pathways.

In this context, it is interesting that the peculiar and rare epilepsy syndrome EIMFS has been reported in patients with biallelic *SLC12A5* variants.^42^ The *SLC12A5* gene encodes for the KCC2 transporter required to maintain the chloride gradient that would otherwise render GABAergic currents excitatory.^43^ Although chloride transport dynamics are exceptionally complex in neural networks, Cl^−^ fluorophores suggest that mature neurons have a median internal chloride concentration between 6 and 14 mM that can reach as high as 40 mM in sensory afferent neurons that do not express the KCC2 transporter.^44,45^ Rapid bursts of synaptic inputs can increase intracellular chloride concentrations in dendritic compartments by as much as 20 mM, which is then extruded by KCC2 at a rate in the order of 5 mM/s.^46^ The precise point at which gain-of-function GABA_A_ receptors will overload the KCC2 extrusion capacity and reverse the chloride conductance is unclear, and computational models suggest it is likely to be highly dynamic with different sensitivities in different cellular compartments.^47^ Nevertheless, combinations of increased GABA sensitivity and decreased desensitization at equilibrium may overload chloride efflux via KCC2 transporters, leading to overlapping characteristic of EIFMS at *SLC12A5* and *GABRB3* variants. The overlap of different phenotypic traits with genetic variants in GABAergic pathways, including different GABA_A_ receptor genes and the transporter KCC2, show that delineating the contributions of GABAergic activity, including chloride reversal potentials, tonic and phasic currents will likely prove essential to understanding how individual phenotypic traits develop and lead to effective treatment.

### Limitations of the study

More precise details of how variants alter desensitization kinetics can be elucidated via kinetic modeling after determining the fast and slow components of desensitization.^32^ In several cases, including the WT and variants such as p.(Ser254Phe), decay currents for individual oocytes did not fit to a two-phase decay, limiting the interpretations we can make. However, the weighted tau values changed in a predictable manner, with the slow time course of desensitization being most likely to be affected. This agrees with the proposal that the slow component of current decay is susceptible to structural rearrangements of the β-subunit. The finer details of how the different structures, particularly the coupling loops and the M1 regions, will be important in understanding how variants in these regions will alter receptor function and how it relates to clinical severity. Another limitation of this study is that the conclusions were drawn from non-neuronal cells, and the findings may be different from the neuronal milieu, or more complicated by the dynamic nature of neuronal GABA_A_ receptor activation, membrane potentials and ionic gradients.

## Conclusion

We have identified altered desensitization properties at *GABRB3* variant receptors that increase desensitization via accelerated current decay or decrease desensitization via increased steady-state currents. We propose that changes in desensitization associates with the severity of the clinical phenotype as a secondary factor but does not define the variants as gain- or loss-of-function. The increased severity of the phenotypes at patients with transmembrane region variants appears to be associated with increased steady state currents, a correlation that may enable more accurate diagnosis.

## Supplementary Material

awad285_Supplementary_Data

## Data Availability

De-identified data will be made available to those eligible. This includes the *GABRB3* database and data used for all analysis in the manuscript. Data will be stored for a minimum of 7 years.
