# Supplementary material for: Correlations of receptor desensitization of gain-of-function GABRB3 variants with clinical severity
Source: Brain. 2023 Aug 30;147(1):224–39. doi: 10.1093/brain/awad285 (PMC10766243; doi:10.1093/brain/awad285)
Supplement: awad285_Supplementary_Data [file awad285_supplementary_data.pdf]

Supplementary Information

Correlations of receptor desensitization of gain-of-function *GABRB3* variants with clinical severity

Susan X.N. Lin[1], Philip K. Ahring[1], Angelo Keramidas[2], Vivian W.Y. Liao[1], Rikke S. Møller[3,4], Mary Chebib[1] and Nathan L. Absalom[1,5*].

[1]Brain and Mind Centre, School of Medical Sciences, Faculty of Medicine and Health, The University of Sydney, Sydney, New South Wales, Australia.
[2]Institute for Molecular Bioscience, The University of Queensland, Saint Lucia, QLD 4072, Australia
[3]Department of Epilepsy Genetics and Personalized Medicine, Danish Epilepsy Centre, Dianalund, Denmark. Member of ERN, EpiCare.
[4]Department of Regional Health Research, Faculty of Health Sciences, University of Southern Denmark
[5]School of Science, University of Western Sydney, Sydney, New South Wales, Australia

Table of Contents

Supplementary Results Text **2**
Concatenated vs binary and ternary freely expressing receptors 2
Simulated synaptic current activation 2
Supplementary Figures **3**
Supplementary Figure S1. Concatenated receptors eliminate recordings from binary α1β3 receptors and display similar maximum currents to free subunits expressed in *Xenopus* oocytes. 3
Supplementary Figure S2. $\beta3^{T287I}$ and $\gamma2^{R323Q}$ variants alter activation and deactivation properties of α1β3γ2 $GABA_A$ receptors expressed in HEK293 cells similarly to receptors expressed in *Xenopus* oocytes. 4
Supplementary Figure S3. Maximum current levels of *GABRB3* gain of function receptors. 5
Supplementary Tables **6**
Table S1. Parameters for concatenated and untethered receptors. 6
Table S2. ANOVA results for Table S1 7
Table S3. Peak currents and I_{ss}/I_{peak} values. 8
Table S4. ANOVA results for Tables 1, S3 and S9. 9
Table S5. Parameters for two-phase current decay. 10
Table S6. ANOVA results for Table S5. 11
Table S7. Parameters of receptors expressed in HEK293 cells. 12
Table S8. ANOVA results for Table S7 13
Table S9. Desensitization parameters of loss-of-function *GABRB3* variants. 14

Supplementary Results Text

Concatenated vs binary and ternary freely expressing receptors

We evaluated the maximum currents, maximal estimated open probability, 3mM GABA current decay rates and steady-state currents at equilibrium and GABA sensitivity for oocytes injected with concatenated α1β3γ2, α1 and β3, or α1, β3 and γ2 subunits. Binary α1β3 receptors displayed markedly higher steady-state currents and steady-state open probability, and higher GABA sensitivity to either concatenated or α1β3γ2 receptors expressed from free subunits (Supplementary Figure S1; Table S1-2). Although broadly similar to α1β3γ2 receptors expressed from free subunits, concatenated receptors had lower GABA sensitivity and decelerated decay rates with the fraction of the slow phase of current decay increased at concatenated receptors (Supplementary Table S1-2), as expected for currents elicited by a lower effective GABA concentration. Thus, the experimental paradigm where concatenated variant receptors were compared to wild-type variants was considered preferable to comparing variants from free subunits, where contamination of α1β3 receptor populations will lead to aberrant results.

Simulated synaptic current activation

Synaptic currents were simulated by brief application (1-2 ms) of 3 mM GABA onto cells or membrane patches (Supplementary Figure S2). The rate of current onset and decay (deactivation) was determined by fitting the onset phase to a single exponential function and the deactivation to two exponential functions. The activation rates for WT and $\gamma2^{R323Q}$-containing receptors were similar. By contrast, $\beta3^{T287I}$-containing receptors activated more slowly than the other two receptors (Supplementary Figure S2, Supplementary Table S7-8). Current deactivation between WT and $\gamma2^{R323Q}$-containing receptors was similar. The weighted deactivation times for the $\beta3^{T287I}$-containing receptors displayed considerably greater variance than the other two experiments, but always greater than the other two receptors (Supplementary Figure S2). However, the mean deactivation time did not reach statistical significance (Supplementary table S7-8).

Supplementary Figures

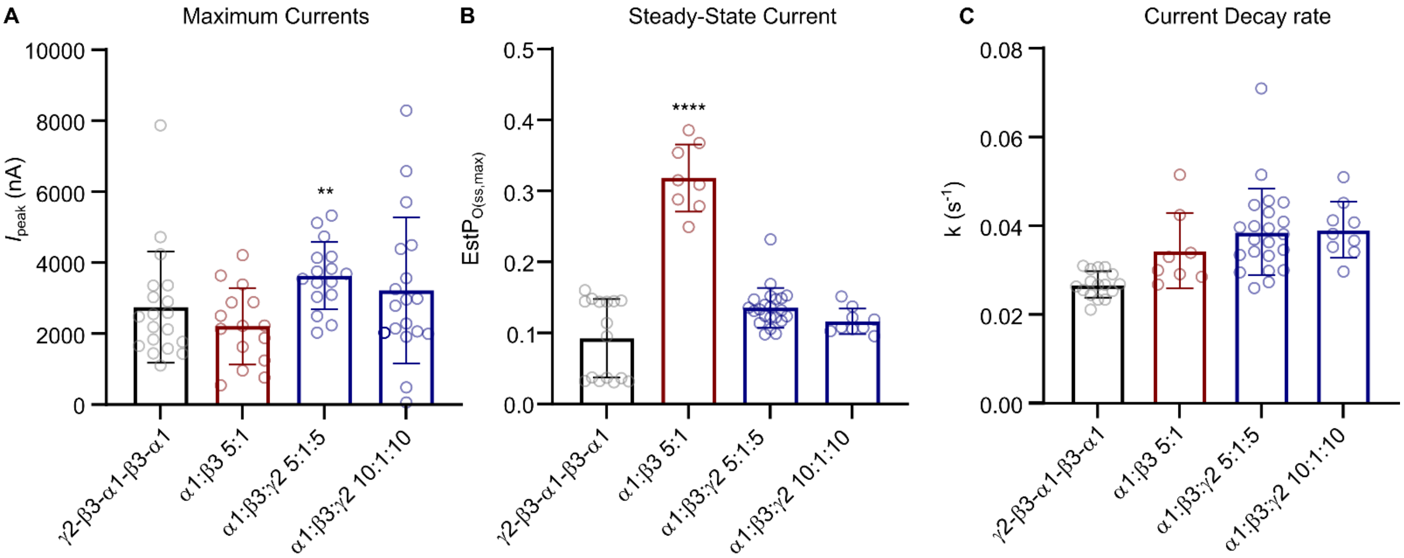

Supplementary Figure S1. Concatenated receptors eliminate recordings from binary α1β3 receptors and display similar maximum currents to free subunits expressed in *Xenopus* oocytes. A Bar graph representing the peak currents elicited by 3 mM GABA at receptors expressed in *Xenopus* oocytes with γ2-β3-α1-β3-α1 concatenated constructs (black), α1 and β3 subunits in a 5:1 ratio (red) and α1, β3 and γ2 subunits in a 5:1:5 or 10:1:10 ratio (blue). **B** Bar graph representing the estimated steady-state open probability elicited by 3 mM GABA at receptors expressed in *Xenopus* oocytes with γ2-β3-α1-β3-α1 concatenated constructs (black), α1 and β3 subunits in a 5:1 ratio (red) and α1, β3 and γ2 subunits in a 5:1:5 or 10:1:10 ratio (blue). **C** Bar graph representing the current decay rate elicited by 3 mM GABA at receptors expressed in *Xenopus* oocytes with γ2-β3-α1-β3-α1 concatenated constructs (black), α1 and β3 subunits in a 5:1 ratio (red) and α1, β3 and γ2 subunits in a 5:1:5 or 10:1:10 ratio (blue). For all graphs, bars represent mean ± s.d, circles represent individual experiments, * $p < 0.05$, ** $p < 0.01$, *** $p < 0.001$, **** $p < 0.0001$ compared to WT, non-parametric ANOVA with Dunn's post-hoc test.

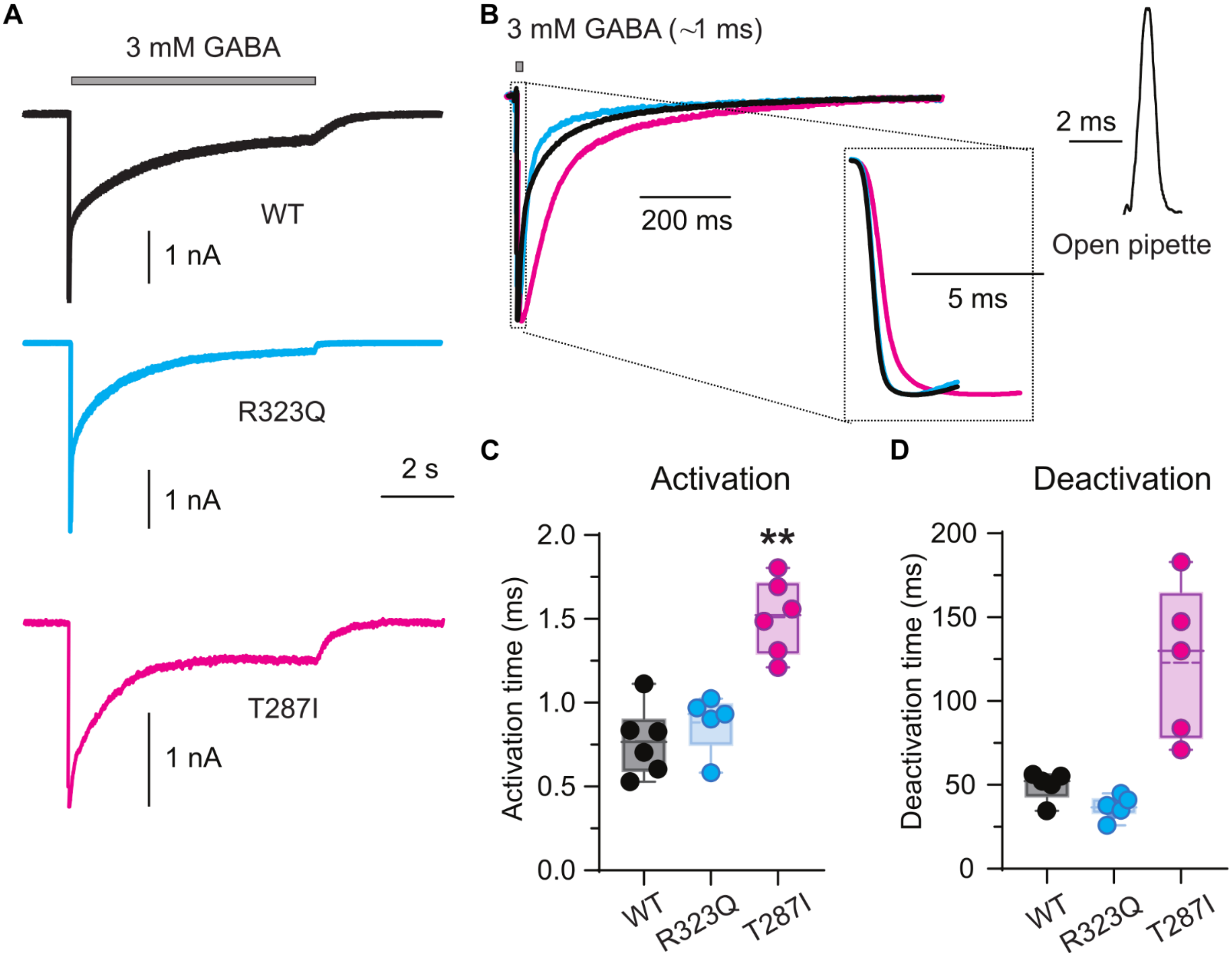

Supplementary Figure S2. β3^{T287I} and γ2^{R323Q} variants alter activation and deactivation properties of α1β3γ2 GABA$_A$ receptors expressed in HEK293 cells similarly to receptors expressed in *Xenopus* oocytes. A Representative traces of WT (black), γ2^{R323Q} (aqua) and β3^{T287I} (pink) variant receptors activated by a prolonged (~7s) 3 mM GABA pulse and recorded from transfected HEK293 cells in whole-cell configuration. **B** Representative traces for determination of activation and deactivation time constants at WT (black), γ2^{R323Q} (aqua) and β3^{T287I} (pink) variant receptors recorded from transfected HEK293 cells. An inset displaying the traces during the activation time is shown, and the open tip potential for a representative wild-type experiment. **C** Box and whisker plot of activation times. **D** Box and whisker plot of deactivation times. For all graphs, bars represent mean ± s.d, circles represent individual experiments, * $p < 0.05$, ** $p < 0.01$, *** $p < 0.001$, **** $p < 0.0001$ compared to WT, non-parametric ANOVA with Dunn's post-hoc test.

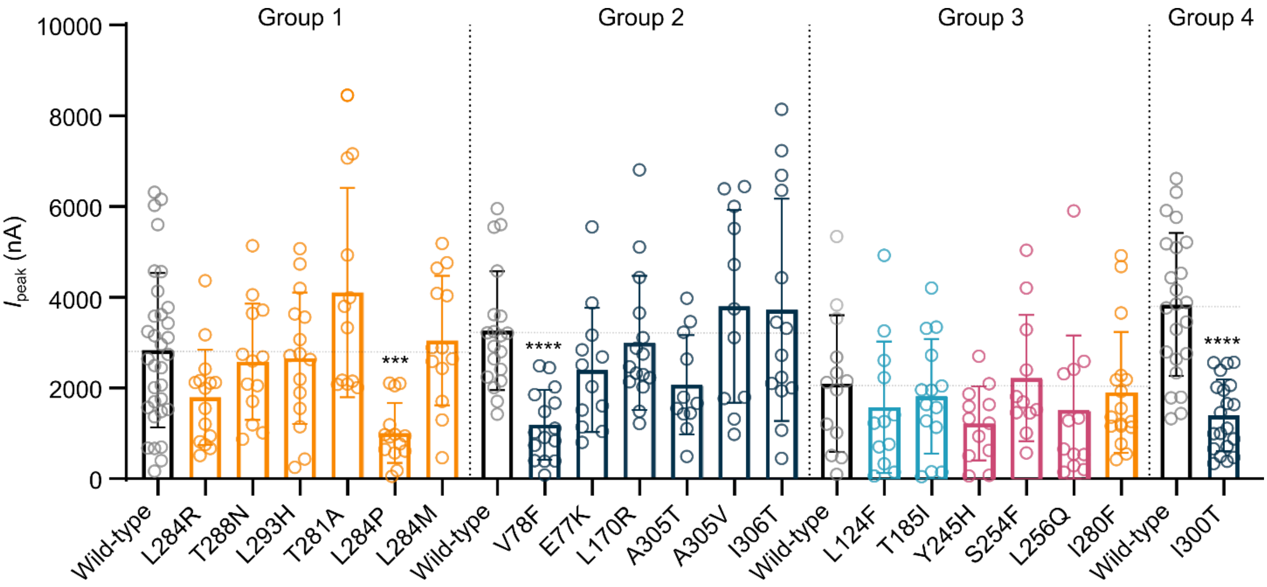

Supplementary Figure S3. Maximum current levels of *GABRB3* gain of function receptors. Bar graph representing the peak currents elicited by 3 mM GABA at wild-type (black) and variant receptors in the extracellular regions (light blue), coupling regions (dark blue), M1 (magenta) and M2 (orange) expressed in *Xenopus* oocytes with γ2-β3-α1-β3-α1 concatenated constructs. Open circles represent individual data points. Bars represent mean ± s.d, circles represent individual experiments, * $p < 0.05$, ** $p < 0.01$, *** $p < 0.001$, **** $p < 0.0001$ compared to WT, non-parametric ANOVA with Dunn's post-hoc test.

Supplementary Tables

Table S1. Parameters for concatenated and untethered receptors.

Construct	Activation properties									
	I_{peak}	p-value[b]	Est $P_{O(max)}$	p-value[b]	N	$logEC_{50}$	p-value[a]	n_H	p-value[b]	n[c]
γ2-β3-α1-β3-α1[a]	2749±1572		0.83±0.07		19	-3.48±0.13		1.6±0.2		11
α1β3 (5:1)	2208±1076	0.99	0.74±0.20	0.99	15	-5.06±0.4	<0.0001	0.56±0.19	<0.0001	8
α1β3γ2 (5:1:5)	3631±946	0.02	1.22±0.11	0.02	16	-4.87±0.25	<0.0001	1.2±0.3	0.09	10
α1β3γ2 (10:1:10)	3222±2057	0.98	1.26±0.43	0.02	18	-4.81±0.33	<0.0001	1.4±0.5	0.99	9
	One-phase decay									
	k	p-value[b]	$EstP_{O(ss,max)}$	p-value[b]	I_{ss}		p-value[b]	I_{ss}/I_{peak}	p-value[b]	n[c]
γ2-β3-α1-β3-α1[a]	0.027±0.003		0.092±0.06		381±162			0.077±0.046		16
α1β3 (5:1)	0.034±0.009	0.068	0.32±0.05	<0.0001	624±116		0.019	0.24±0.04	<0.0001	8
α1β3γ2 (5:1:5)	0.039±0.010	<0.0001	0.14±0.03	0.25	640±171		0.0006	0.17±0.03	<0.0001	22
α1β3γ2 (10:1:10)	0.039±0.006	0.0002	0.12±0.02	0.99	713±202		0.0009	0.15±0.02	0.0174	9
	Two-phase decay									
	$Fraction_{fast}$	p-value[b]	$\tau_{weighted}$	p-value[b]	τ_{fast}		p-value[b]	τ_{slow}	p-value[b]	n[c]
γ2-β3-α1-β3-α1[a]	0.25±0.04		40.2±5.6		9.95±5.0			50.5±6.8		16
α1β3 (5:1)	0.48±0.08	0.0001	43.5±13	0.999	10.3±3.1		n.d.*	73.4±17	0.02	7
α1β3γ2	0.46±0.09	<0.0001	31.6±8.0	0.0196	7.36±2.4		n.d.*	53.1±11	0.99	18
α1β3γ2 (10:1:10)	0.45±0.07	<0.0004	29.1±4.9	0.0067	7.7±2.7		n.d.*	46.9±8.7	0.75	8

[a]Experimental groups where variants and wild-type performed on same day
[b]One-way Kruskal-Wallis non-parametric ANOVA followed by a Dunn's post-hoc test except $logEC_{50}$ where a one-way ANOVA with Dunnett's post-hoc test
[c]Numbers of individual oocytes where two-phase decay successfully fitted
*Kruskal-Wallis test p>0.05, Dunn's post-hoc test not performed

Table S2. ANOVA results for Table S1

Activation properties											
I_{peak}			Est $P_{O(max)}$			$logEC_{50}$			n_H		
H[a]	df[b]	p	H[a]	df[b]	p	F[c]	df[d]	p	H[a]	df[b]	p
11.75	3	0.008	18.83	3	0.003	68.68	3, 34	<0.0001	21.1	3	0.0001
One-phase decay											
k			Est$P_{O(ss,max)}$			I_{ss}			I_{ss}/I_{peak}		
H[a]	df[b]	p	H[a]	df[b]	p	H[a]	df[b]	p	H[a]	df[b]	p
25.21	3	<0.0001	23.82	3	<0.0001	19.24	3	0.0002	39.86	3	<0.0001
$Fraction_{fast}$			$\tau_{weighted}$			τ_{fast}			τ_{slow}		
H[a]	df[b]	p	H[a]	df[b]	p	H[a]	df[b]	p	H[a]	df[b]	p
31.6	3	<0.0001	15.88	3	0.0012	6.892	3	0.075	12.09	3	0.0071

[a]H is the Kruskal-Wallis statistic
[b]df is the degrees of freedom
[c]The F-statistic from the one-way ANOVA
[d]df is the degrees of freedom of the ANOVA

Table S3. Peak currents and I_{ss}/I_{peak} values.

Variant	I_{peak} (nA)	p-value[a]	I_{ss} (nA)	p-value[a]	I_{ss}/I_{peak}	p-value[a]	n[b]
Group 1[c]							
Wild-type	2992±1342		581±486		0.18±0.08		28
T287I	3272±1439	n.d.*	911±542	0.03	0.30±0.09	<0.0001	22
γ2(R323Q)	2597±1136	n.d.*	432±243	0.78	0.16±0.04	0.45	16
Group 2[d]							
Wild-type	2832±1701		359±216		0.13±0.04		32
T281A	4102±2307	0.68	811±584	0.01	0.19±0.04	0.10	12
L284M	3045±1427	0.99	372±186	0.99	0.13±0.04	0.99	13
L284P	1006±660	0.0009	343±232	0.99	0.36±0.12	<0.0001	14
L284R	1793±1051	0.32	754±778	0.19	0.38±0.14	<0.0001	15
T288N	2576±1283	0.99	289±213	0.99	0.10±0.03	0.63	13
L293H	2655±1450	0.99	256±175	0.91	0.09±0.03	0.20	15
Group 3[e]							
Wild-type	3262±1311		420±234		0.12±0.03		19
E77K	2396±1366	0.59	298±279	0.38	0.12±0.04	n.d.*	12
V78F	1187±770	<0.0001	99±57	<0.0001	0.11±0.09	n.d.*	15
L170R	2994±1477	0.99	338±144	0.99	0.12±0.04	n.d.*	15
A305T	2070±1096	0.20	245±116	0.38	0.12±0.03	n.d.*	11
A305V	3798±2125	0.99	600±486	0.99	0.14±0.05	n.d.*	11
I306T	3723±2451	0.99	518±407	0.99	0.14±0.05	n.d.*	14
Group 4[f]							
Wild-type	2094±1505		246±201		0.11±0.03		13
L124F	1575±1449	n.d.*	179±192	n.d.*	0.10±0.06	0.99	12
T185I	1819±1263	n.d.*	206±218	n.d.*	0.09±0.04	0.99	14
Y245H	1220±815	n.d.*	242±204	n.d.*	0.19±0.05	0.04	12
S254F	2220±1390	n.d.*	483±354	n.d.*	0.21±0.07	0.02	11
L256Q	1516±1644	n.d.*	393±585	n.d.*	0.19±0.10	0.15	12
I280F	1904±1334	n.d.*	169±159	n.d.*	0.09±0.06	0.75	17
Group 5[g]							
Wild-type	5820±2265		887±336		0.16±0.05		16
M80K	3137±1246	0.001	274±142	<0.0001	0.09±0.03	0.003	16
Q249K	5928±1330	0.99	832±195	0.99	0.14±0.04	0.99	16
T281I	5912±2206	0.99	228±94	<0.0001	0.04±0.01	<0.0001	16
Y302C	4222±1367	0.31	970±246	0.99	0.24±0.04	0.17	11
Wild-type	3839±1580		539±197		0.15±0.03		23
I300T	1393±788	<0.0001	44±26	<0.0001	0.04±0.02	<0.0001	20

[a]One-way Kruskal-Wallis non-parametric ANOVA followed by a Dunn's post-hoc test except I300T that was compared with a Mann-Whitney U test, experimental groups compared separately
[b]Numbers of individual oocytes to determine k, Est $P_{O(max)}$ and Est $P_{O(ss, max)}$
[c-g]Experimental groups where variants and wild-type performed on same day
*n.d. Not determined as $p > 0.05$ for initial ANOVA

Table S4. ANOVA results for Tables 1, S3 and S9.

Table 1 Values	k			Est $P_{O(ss)}$			Est $P_{O(max)}$		
Group	H[a]	df[b]	p	H[l]	df[b]	p	H[l]	df[b]	p
Group 1	21.83	2	<0.0001	38.09	2	<0.0001	21.28	2	<0.0001
Group 2	11.89	6	0.0645	81.69	6	<0.0001	15.15	6	0.0191
Group 3	53.69	6	<0.0001	14.02	6	0.0294	11.65	6	0.0702
Group 4	66.04	6	<0.0001	43.1	6	<0.0001	10.22	6	0.1156
Group 5[c]	39.34	5	<0.0001	72.47	5	<0.0001	47.17	5	<0.0001
Group 5[d]	50.92	4	<0.0001	75.31	4	<0.0001	38.89	4	<0.0001
Table S3 Values	I_{peak}			I_{ss}			I_{ss}/I_{peak}		
Group	H[a]	df[b]	p	H[l]	df[b]	p	H[l]	df[b]	p
Group 1	2.30	2	0.3163	9.70	2	0.0078	25.99	2	<0.0001
Group 2	27.68	6	0.0001	24.18	6	0.0005	77.56	6	<0.0001
Group 3	26.97	6	00001	35.2	6	<0.0001	10.84	6	0.0933
Group 4	5.279	6	0.5085	10.22	6	0.1157	43.65	6	<0.0001
Group 5[c]	38[e]	-	-	0[e]	-	-	0[e]	-	-
Group 5[d]	24.68	4	<0.0001	53.28	4	<0.0001	61.16	4	<0.0001

[a]H is the Kruskal-Wallis statistic
[b]df is the degrees of freedom
[c]For 3 mM GABA applications
[d]For 30 mM GABA applications
[e]Mann-Whitney U statistic, only two groups to compare

Table S5. Parameters for two-phase current decay.

Variant	$Fraction_{fast}$	p-value[a]	$\tau_{weighted}$	p-value[a]	τ_{fast}	p-value[a]	τ_{slow}	p-value[a]	n[b]
Group 1[c]									
Wild-type	0.16±0.08		64±15		8.2±3.7		75±17		23
T287I	0.26±0.10	0.018	60+17	0.30	7.7±3.7	0.59	78±21	0.70	14
Group 2[d]									
Wild-type	0.21±0.17		33±10		8.0±6.3		39±13		15
T281A	0.26±0.13	0.99	26±6	0.70	4.8±3.8	n.d.*	33±8	0.99	8
L284M	0.05±0.03	0.12	29±6	0.99	4.7±3.0	n.d.*	64±30	0.99	8
L284P	0.36±0.12	0.01	52±24	0.10	6.3±2.5	n.d.*	78±36	0.001	14
L284R	0.36±0.15	0.03	42±19	0.75	5.9±4.0	n.d.*	64±30	0.07	12
T288N	0.29±0.18	0.99	36±1	0.99	9.0±6.5	n.d.*	47±12	0.77	13
L293H	0.28±0.11	0.64	33±1	0.99	7.2±3.1	n.d.*	43±11	0.99	13
Group 3[e]									
Wild-type	0.19±0.12		36±6		6.8±3.9		43±9		12
E77K	0.40±0.27	0.07	20±4	<0.0001	8.7±5.9	n.d.*	32±17	0.003	11
V78F	0.27±0.09	0.99	28±4	0.71	8.3±4.9	n.d.*	38±9	0.99	11
L170R	0.23±0.07	0.99	35±11	0.99	7.6±3.9	n.d.*	43±12	0.99	9
A305T	0.35±0.07	0.004	22±4	0.0001	7.1±2.6	n.d.*	30±6	0.02	11
A305V	0.29±0.08	0.13	23±3	0.0013	7.0±2.7	n.d.*	29±4	0.004	11
I306T	0.17±0.12	0.99	31±11	0.54	7.9±3.4	n.d.*	36±11	0.35	9
Group 4[f]									
Wild-type	0.22±0.13		33±9		6.5±5.9		40±10		15
L124F	0.23±0.13	0.99	25±3	0.33	6.9±4.0	0.99	31±4	0.55	10
T185I	0.28±0.13	0.97	28±5	0.99	7.0±4.0	0.99	38±15	0.99	10
Y245H	0.14±0.02	0.61	71±20	0.03	13.6±2.4	0.0045	94±20	0.009	10
S254F	0.18±0.1	0.99	20±4	0.07	5.8±3.0	0.99	23±3	0.10	4
L256Q	0.41±0.1	0.007	35±9	0.99	11.1±4.6	0.08	51±13	0.96	12
I280F	0.14±0.08	0.85	17±4	0.0003	1.8±1.2	0.07	20±4	0.0008	10
Group 5[g]									
Wild-type	0.23±0.01		53±9		11.1±4.3		66±11		13
I300T	0.13±0.04	0.08	38±7	0.003	10.4±1.7	0.85	42±7	0.002	5

[a]Mann-Whitney test for Groups 1 and 5, one-way Kruskal-Wallis non-parametric ANOVA followed by a Dunn's post-hoc test for Groups 2-4, experimental groups compared separately
[b]Numbers of individual oocytes where two-phase decay successfully fitted
[c-g]Experimental groups where variants and wild-type performed on same day

Table S6. ANOVA results for Table S5.

	$Fraction_{fast}$			$\tau_{weighted}$			τ_{fast}			τ_{slow}		
Group	H[a]	df[b]	p	H[1]	df[b]	p	H[1]	df[b]	p	H[1]	df[b]	p
Group 1	64[c]	-	-	127[c]	-		143[c]	-	-	148[c]	-	-
Group 2	31.93	6	<0.0001	18.19	6	0.0058	6.72	6	0.35	30.43	6	<0.0001
Group 3	22.04	6	0.0012	40.77	6	<0.0001	1.95	6	0.92	26.64	6	0.0002
Group 4	29.53	6	<0.0001	51.06	6	<0.0001	36.29	6	<0.0001	55.82	6	<0.0001
Group 5	14[c]	-	-	4[c]	-	-	14[c]	-	-	3[c]	-	-

[a]H is the Kruskal-Wallis statistic
[b]df is the degrees of freedom
[c]The Mann-Whitney U-statistic is given where there is only one variant in the group

Table S7. Parameters of receptors expressed in HEK293 cells.

	Deactivation (1-2ms application of GABA)							
Construct	Activation (ms)	p-value	$\tau_{weighted}$	p-value	$Fraction_{fast}$	τ_{fast}	τ_{slow}	n
$\alpha1+\beta3+\gamma2$	0.77±0.21		49.7±8.8		66.6±16.2	8.9±1.9	174±115	6
$\alpha1+\beta3+\gamma2^{R323Q}$	0.88±0.17	0.94	36.7±6.5	0.41	69.4±7.2	7.2±3.5	105±23	6
$\alpha1+\beta3^{T287I}+\gamma2$	1.51±0.22	0.002	123±46	0.09	86.9±10.6	44±9.4	965±1110	7
	Desensitization (Prolonged application of GABA)							
	I_{ss}/I_{peak}	p-value	$\tau_{weighted}$	p-value	$Fraction_{fast}$	τ_{fast}	τ_{slow}	n
$\alpha1+\beta3+\gamma2$	0.208±0.047		1860±183		18.5±7	229±214	2237±312	5
$\alpha1+\beta3+\gamma2^{R323Q}$	0.087±0.04	0.015	1347±264	0.11	19.3±10	147±117	1628±197	5
$\alpha1+\beta3^{T287I}+\gamma2$	0.270±0.066	0.50	2361±271	0.28	31.8±17	195±113	3392±383	5

Table S8. ANOVA results for Table S7

Deactivation (1-2ms application of GABA)						Desensitization (Prolonged application of GABA)					
Activation (ms)			$\tau_{weighted}$			I_{ss}/I_{peak}			$\tau_{weighted}$		
H[a]	df[b]	P	H[a]	df[b]	P	H[a]	df[b]	P	H[a]	df[b]	P
11.52	2	0.0002	11.32	2	0.0002	15.93	3	<0.0001	11.58	2	<0.0001

[a]H is the Kruskal-Wallis statistic
[b]df is the degrees of freedom

Table S9. Desensitization parameters of loss-of-function *GABRB3* variants.

Variant[a]	$\Delta LogEC_{50}$[b]	k ± s.d.	p-value[c]	Est $P_{O(ss,max)}$ ± s.d.	p-value[c]	Est $P_{O(max)}$ ± s.d.	p-value[c]	n[d]
Group 5								
Wild-type		0.031 ± 0.0086		0.15 ± 0.043		1.1 ± 0.19		14
M80K	-0.56	0.049 ± 0.011	<0.0003	0.049 ± 0.013	<0.0001	0.56 ± 0.13	<0.0001	13
Q249K	-0.53	0.029 ± 0.0076	>0.9999	0.13 ± 0.034	>0.9999	0.86 ± 0.25	0.1424	10
T281I	-0.55	0.043 ± 0.010	0.0170	0.036 ± 0.0095	<0.0001	0.97 ± 0.22	>0.9999	11
Y302C	-0.81	0.023 ± 0.0080	0.3327	0.16 ± 0.026	>0.9999	0.68 ± 0.19	<0.0001	13

[a]Unless otherwise specified, variants are in the β3 subunit
[b]Values taken from Absalom et al, 2022.
[c]One-way Kruskal-Wallis non-parametric ANOVA followed by a Dunn's post-hoc test, experimental groups compared separately
[d]Numbers of individual oocytes to determine k, Est $P_{O(max)}$ and Est $P_{O(ss, max)}$
[*]n.d. Not determined as p > 0.05 for initial ANOVA
